# Computational Study of Growth and Remodeling in Ascending Thoracic Aortic Aneurysms Considering Variations of Smooth Muscle Cell Basal Tone

**DOI:** 10.3389/fbioe.2020.587376

**Published:** 2020-11-03

**Authors:** Ataollah Ghavamian, S. Jamaleddin Mousavi, Stéphane Avril

**Affiliations:** Mines Saint-Etienne, Université Lyon, Université Jean Monnet, INSERM, U 1059 Sainbiose, Centre CIS, Saint-Étienne, France

**Keywords:** finite element, constrained mixture theory, growth and remodeling, smooth muscle cells, active stresses

## Abstract

In this paper, we investigate the progression of Ascending Thoracic Aortic Aneurysms (ATAA) using a computational model of Growth and Remodeling (G&R) taking into account the composite (elastin, four collagen fiber families and Smooth Muscle Cells—SMCs) and multi-layered (media and adventitia) nature of the aorta. The G&R model, which is based on the homogenized Constrained Mixture theory, is implemented as a UMAT in the Abaqus finite-element package. Each component of the mixture is assigned a strain energy density function: nearly-incompressible neo-Hookean for elastin and Fung-type for collagen and SMCs. Active SMCs tension is additionally considered, through a length-tension relationship having a classic inverted parabola shape, in order to investigate its effects on the progression of ATAA in a patient-specific model. A sensitivity analysis is performed to evaluate the potential impact of variations in the parameters of the length-tension relationships. These variations reflect in variations of SMCs normal tone during ATAA progression, with active stress contributions ranging between 30% (best case scenario) and 0% (worst case scenario) of the total wall circumferential stress. Low SMCs active stress in the worst case scenarios, in fact, affect the rates of collagen deposition by which the elastin loss is gradually compensated by collagen deposition in the simulated ATAA progression, resulting eventually in larger aneurysm diameters. The types of length-tension relationships leading to a drop of SMCs active stress in our simulations reveal a critical condition which could also result in SMCs apoptosis.

## 1. Introduction

Nowadays, the paramount importance of mechanobiology is widely acknowledged in morphogenesis and pathogenesis (Humphrey, 2008). The adaptation ability of soft tissues relies on the existence of a preferred load-bearing mechanical state, the so-called homeostasis, across multiple length/time scales. At the tissue scale, this is exhibited through continuous mass changes of the components of the Extracellular Matrix (ECM), such as elastin, collagen, and proteoglycans in blood vessels (Humphrey and Rajagopal, 2002; Cyron et al., 2016). Mechanobiology implies that Growth and Remodeling (G&R) of the biological tissues is mediated by mechanical stresses (Grossman, 1980; Humphrey, 2008).

In order to numerically simulate G&R in arteries, the constrained mixture model (CMM) was developed by Humphrey and Rajagopal (2002) and, thereafter, has been increasingly utilized (Watton et al., 2004; Baek et al., 2006; Zeinali-Davarani and Baek, 2012; Valentín et al., 2013; Cyron et al., 2016; Braeu et al., 2017; Lin et al., 2017; Famaey et al., 2018; Latorre and Humphrey, 2018b). Baek et al. (2006) proposed a two-dimensional CMM for arterial G&R. A three-dimensional representative straight cylindrical artery was also introduced by Karsaj et al. (2010) with evolving geometry, structure, and mechanical properties under changes in hemodynamics (i.e., mean blood flow/pressure). Valentín et al. (2013) established a non-linear model using the Finite Element Method (FEM) on the basis of the Constrained Mixture Theory (CMT) aimed at facilitating numerical analyses of arterial adaptation and mal-adaptation. They could predict changes in fiber orientations and quantities, degradation of elastin and loss of Smooth Muscle Cells (SMCs), including disease progression and evolving geometries. Famaey et al. (2018) implemented the same model in Abaqus (Hibbit et al., 2011) and predicted adaptation of a pulmonary autograft. Cyron et al. (2016), and then Braeu et al. (2017), introduced the homogenized CMM framework for G&R using an informal temporal averaging approach, bringing simplicity and computational efficiency. Lin et al. (2017) combined homogenization and the CMT to simulate the dilatation of abdominal aortic aneurysms. Their methodology could capture important aspects, such as mass turnover in arterial walls with a low computational cost. Recently, Latorre and Humphrey (2018a) introduced a new rate-based CMM formulation suitable for studying mechanobiological equilibrium and stability of soft tissues exposed to transient or sustained changes, permitting direct resolution of G&R problems with a quasi-static approach.

Despite the significant insight of the CMM methodology in arterial G&R, it has generally been employed in the case of canonical problems (Baek et al., 2006; Cyron et al., 2016; Laubrie et al., 2020) or single-layer thick-wall axisymmetric (Braeu et al., 2017; Lin et al., 2017) approximations. The extension to ATAAs was challenging due to simultaneous and region-specific evolutions of geometry, material properties (Farzaneh et al., 2019), and hemodynamic loads (Humphrey and Holzapfel, 2012; Condemi et al., 2017). Only recently, Mousavi et al. (2019) proposed a non-linear FEM solution based on the homogenized CMT to simulate G&R in a layer-specific and patient-specific ATAA. They showed that elastin loss leads to a transfer of stress to the adventitia and continuous adaptation of the stress distribution through changes in ATAA shape.

Nevertheless, the significant role of SMCs in regularizing the wall tension of hollow organs should not be neglected. This occurs through contraction and relaxation of SMCs which is regulated by phosphorylation of the myosin motors associated with the smooth muscle contractile units. SMCs also help to control the arterial wall stiffness (e.g., during a cardiac cycle) and, then, regularizing the blood pressure (Murtada et al., 2010b). It is reported by Murtada and Holzapfel (2014) that the large elastic deformations of arteries undergoing a physiological loading cannot rely only on a passive response. The active contribution (contractility) of SMCs can generate significant tension. Rachev and Hayashi (1999) proposed a phenomenological model for SMCs in the arterial wall with a length-tension relationship depending on the circumferential stretch. Similarly, Schmitz and Böl (2011) proposed a steady-state model of SMCs activation containing a phenomenological explanation of the active length–tension behavior. Zulliger et al. (2004) extended this method, but this time for a vascular SMCs. They investigated the pressure-radius curve for three different SMC states (i.e., fully relaxed, maximally contracted, normal tone) under physiological and pathological conditions with varying levels of SMCs tone. Standley et al. (2002) proposed a thermodynamically consistent constitutive model as a function of stretches, a variable accounting for the SMC state (free unphosphorylated myosin, phosphorylated cross-bridges, phosphorylated and dephosphorylated cross-bridges attached to actin), a variable related to the Ca^2+^ concentration, and temperature. They illustrated how SMCs contraction and related stretch are co-dependent. This work inspired Murtada et al. (2010a) to propose a structural description of the SMCs contractile unit including elastic elongation of the cross-bridges (Hai and Murphy, 1988; Yang et al., 2003) and myofilament sliding. They showed that the driving stress, in general, depends on the state of the muscle and, during contraction, depends only on the attached cycling cross-bridges. The model was extended (Murtada et al., 2010a, 2012) to capture the active length-tension relationship, the evolution law of filament sliding and the isotonic force-shortening velocity relationship. Murtada and Holzapfel (2014) studied the role of SMCs in large elastic arteries using a FEM simulation and demonstrated that, yet, changes in the intracellular calcium amplitudes barely affect the circumferential stress. An increase in the mean intracellular calcium value and, then, in the medial wall thickness, clearly results in changes in stress distribution and the overall deformation. Murtada et al. (2015) investigated the active tone of SMCs in the specific case of murine descending thoracic aortas undergoing acute loading changes and showed the variation in active SMCs tone depending on different induced stretches. They realized that the active tone was reduced when the artery adapted below the optimal stretch with no significant change in passive behavior.

Although the aforementioned accomplished contributions have brought a significant insight on the active role of SMCs into soft tissues undergoing physiological loading, there is still a pressing need to have computational models investigating the possible variations of SMCs contractility in realistic multi-layer patient-specific geometries with irregular boundary conditions and complex deformations. In this work, the G&R model developed by Mousavi et al. (2019) is extended to include the variations of SMCs contractility and a sensitivity analysis is performed to evaluate their impact on ATAA progression in a patient-specific model.

## 2. Materials and Methods

### 2.1. Mathematical Model

#### 2.1.1. Kinematics

We consider the three dimensional deformation of a continuum (e.g., *in vivo* healthy configuration of a blood vessel) moving from its initial undeformed configuration, say before any G&R happens, occupying a volume Ω_0_, of boundary ∂Ω_0_, to a time dependent deformed configuration occupying a volume Ω(*t*), of boundary ∂Ω(*t*) at time *t*. The motion of the continuum is defined through a deformation mapping, such as ***x*** = ϕ(***X***, *t*) by which a solid particle at the reference state is transformed to the spatial configuration. The total deformation gradient ***F*** of a mixture, consisting of different constituents (e.g., elastin, collagen fiber families, and SMCs), can be expressed as

(1) $$\begin{array}{l} F\left(X,t\right)=\frac{\partial \phi \left(X,t\right)}{\partial X}. \end{array}$$

The deformation gradient essentially relates a fiber at material configuration to its spatial counterpart (*d****x*** = ***F****d****X***). Additionally, the volume map *J* is defined as

(2) $$\begin{array}{l} J=\text{det}\left(F\right), \end{array}$$

linking a reference volume element into the deformed states (*d*Ω = *Jd*Ω_0_, *J* > 0).

Two main assumptions are made for the deformation gradient on the basis of CMT, namely, (1) all constituents deform together resulting in a unified deformation gradient ***F*** and (2) the deformation gradient of each constituent *i* of the mixture (*i* ∈ {e, c_*j*_, m}, where e stands for elastin, c_*j*_ stands for collagen family *j* where *j* ∈ {1, 2, 3, 4} and m stands for SMCs) is decomposed into an elastic $F_{\text{el}}^{i}$ and an inelastic $F_{\text{gr}}^{i}$ part as

(3) $$\begin{array}{l} F=F_{\text{el}}^{i}F_{\text{gr}}^{i}=F_{\text{el}}^{i}F_{\text{r}}^{i}F_{\text{g}}^{i}, \end{array}$$

where the elastic deformation gradient $F_{\text{el}}^{i}$ is responsible for the generation of the stress field and the inelastic deformation gradient $F_{\text{gr}}^{i}$ considers the differential mass turnover in $F_{\text{g}}^{i}$ and the changes in the microstructure in $F_{\text{r}}^{i}$.

Initially ***F***(*t*_0_) = ***I*** where ***I*** is the identity tensor, each constituent *i* of the mixture has an individual elastic deformation gradient $F_{\text{el}}^{i}\left(t_{0}\right)$ corresponding to its deposition stretch $F_{\text{el}}^{i}\left(t_{0}\right)=G_{\text{h}}^{i}={\left(F_{\text{r}}^{i}\left(t_{0}\right)\right)}^{-1}$ (Mousavi and Avril, 2017) in which $G_{\text{h}}^{i}$ are second order tensors defined, such as

(4a) $$\begin{array}{l} G_{\text{h}}^{\text{e}}=\text{diag}\left[\frac{1}{{\lambda_{0}^{\text{e}}}_{\theta }{\lambda_{0}^{\text{e}}}_{z}},{\lambda_{0}^{\text{e}}}_{\theta },{\lambda_{0}^{\text{e}}}_{z}\right], \end{array}$$

(4b) $$\begin{array}{l} G_{\text{h}}^{i}=\lambda_{0}^{i}a_{0}^{i}⊗a_{0}^{i}+\frac{1}{\sqrt{\lambda_{0}^{i}}}\left(I-a_{0}^{i}⊗a_{0}^{i}\right);\;i\in \left\{{\text{c}}_{j},\text{m}\right\}, \end{array}$$

where ${\lambda_{0}^{\text{e}}}_{\theta }$ and ${\lambda_{0}^{\text{e}}}_{z}$ represent the initial (in the undeformed configuration before any G&R) deposition stretches of elastin in the circumferential and longitudinal directions, respectively, and $\lambda_{0}^{i}$ stands for the initial deposition stretch of constituent *i* in the fiber direction whose unit vector is denoted by $a_{0}^{i}$.

#### 2.1.2. Balance of Linear Momentum

The G&R deformation process is typically governed by the balance of the linear momentum equation

(5) $$\begin{array}{l} \text{DIV}\left(P\right)+\varrho_{0}b_{0}=0, \end{array}$$

in which DIV is the material divergence operator, ***P*** denotes the first Piola-Kirchhoff stress tensor, ***b***_0_ is the body forces per unit mass and $\varrho_{0}=\underset{i}{\sum }\varrho_{0}^{i}$ represents the mass density (per unit undeformed volume) of the mixture, which is equivalent to the sum of mass densities (per unit undeformed volume) of all constituents. Note here that the dynamic effects (i.e., inertia or viscoelasticity) are neglected since the G&R progression occurs at very slow time scales (days to months) (Braeu et al., 2017).

#### 2.1.3. Mechanobiological Constitutive Model

A strain energy density function *W* (per unit undeformed volume) is introduced (Braeu et al., 2017; Mousavi and Avril, 2017)

(6) $$\begin{array}{l} W=\underset{\text{elastin}}{\underset{︸}{\varrho_{0}^{\text{e}}\left(t\right)W^{\text{e}}}}+\underset{\text{Collagen fiber families}}{\underset{︸}{\overset{n}{\underset{j=1}{\sum }}\varrho_{0}^{{\text{c}}_{j}}\left(t\right)W^{{\text{c}}_{j}}}}+\underset{\text{SMCs}}{\underset{︸}{\varrho_{0}^{\text{m}}\left(t\right)W^{\text{m}}}}. \end{array}$$

In the above equation, $\varrho_{0}^{i}\left(t\right)$ and *W*^*i*^ (*i* ∈ {e, c_*j*_, m}) represent, respectively, the mass densities and strain energy densities (per unit mass) of each individual constituent.

A nearly incompressible Neo-Hookean material model is introduced for elastin as (Holzapfel et al., 2000; Mousavi and Avril, 2017)

(7) $$\begin{array}{l} W^{\text{e}}=W^{\text{e}}\left({\bar{I}}_{1}^{\text{e}},J\right)=\underset{W_{\text{Deviatoric}}^{\text{e}}}{\underset{︸}{\frac{\mu^{\text{e}}}{2}\left({\bar{I}}_{1}^{\text{e}}-3\right)}}+\underset{W_{\text{Volumetric}}^{\text{e}}}{\underset{︸}{\frac{\kappa }{2}{\left(J_{\text{el}}^{\text{e}}-1\right)}^{2}}}, \end{array}$$

where μ^e^ and κ stand for the shear modulus and the bulk modulus (stress per unit mass dimensions) of the elastin, respectively. $J_{\text{el}}^{\text{e}}$ denotes the elastic contribution of the Jacobian for elastin and ${\bar{I}}_{1}^{i}$ is the isochoric first invariant of the elastic Cauchy-Green tensor, defined, such as

(8) $$\begin{array}{l} {\bar{I}}_{1}^{\text{e}}=tr\left({\bar{C}}_{\text{el}}^{\text{e}}\right), \end{array}$$

where ${\bar{C}}_{\text{el}}^{\text{e}}={{\bar{F}}_{\text{el}}^{\text{e}}}^{T}{\bar{F}}_{\text{el}}^{\text{e}}$ is the elastic part of the isochoric right Cauchy-Green tensor for elastin. The isochoric deformation gradient is defined as ${\bar{F}}_{\text{el}}^{\text{e}}={\left(J_{\text{el}}^{\text{e}}\right)}^{-1/3}F_{\text{el}}^{\text{e}}$.

The strain energy density of collagen families is described with a Fung-type exponential expression (Mousavi and Avril, 2017)

(9) $$\begin{array}{l} W^{{\text{c}}_{j}}\left(I_{4}^{{\text{c}}_{j}}\right)=\frac{k_{1}^{{\text{c}}_{j}}}{2k_{2}^{{\text{c}}_{j}}}\left[\text{exp}\left(k_{2}^{{\text{c}}_{j}}{\left(I_{4}^{{\text{c}}_{j}}-1\right)}^{2}\right)-1\right], \end{array}$$

where $k_{1}^{{\text{c}}_{j}}$ and $k_{2}^{{\text{c}}_{j}}$ are stress per unit mass and dimensionless material parameters, respectively, taking different values depending whether the fibers are under compression or tension (Bersi et al., 2016; Mousavi and Avril, 2017), and $I_{4}^{{\text{c}}_{j}}$ is the fourth invariant which may be written, such as

(10) $$\begin{array}{l} I_{4}^{{\text{c}}_{j}}=C_{\text{el}}^{{\text{c}}_{j}}:\left(a^{{\text{c}}_{j}}⊗a^{{\text{c}}_{j}}\right)={\left(\lambda_{\text{el}}^{{\text{c}}_{j}}\right)}^{2};\;a^{{\text{c}}_{j}}=\frac{F_{gr}^{{\text{c}}_{j}}a_{0}^{{\text{c}}_{j}}}{\left\|F_{gr}^{{\text{c}}_{j}}a_{0}^{{\text{c}}_{j}}\right\|}, \end{array}$$

where $C_{\text{el}}^{{\text{c}}_{j}}$ is the elastic part of the right Cauchy-Green stretch tensor, $a^{{\text{c}}_{j}}$ is the unit vector along the fiber direction in the inelastically deformed intermediate configuration and $\lambda_{\text{el}}^{{\text{c}}_{j}}$ is the elastic stretch of collagen fibers.

The passive and active strain energy density of SMCs is also introduced as (Wilson et al., 2013; Murtada and Holzapfel, 2014; Murtada et al., 2015)

(11) $$\begin{array}{l} W^{\text{m}}\left(I_{4}^{\text{m}},\lambda_{\text{act}}\right)=\underset{W_{\text{p}ass}^{\text{m}}}{\underset{︸}{\frac{k_{1}^{\text{m}}}{2k_{2}^{\text{m}}}\left[\text{exp}\left(k_{2}^{\text{m}}{\left(I_{4}^{\text{m}}-1\right)}^{2}\right)-1\right]}}\\ \begin{array}{l} \;+\underset{W_{\text{act}}^{\text{m}}}{\underset{︸}{\frac{\sigma_{\text{actmax}}}{\varrho_{0}}\left(\lambda_{\text{act}}+\frac{1}{3}\frac{{\left(\lambda_{\text{max}}-\lambda_{\text{act}}\right)}^{3}}{{\left(\lambda_{\text{max}}-\lambda_{0}\right)}^{2}}\right)}}, \end{array} \end{array}$$

where $k_{1}^{\text{m}}$ and $k_{2}^{\text{m}}$ are similar to the $k_{1}^{{\text{c}}_{j}}$ and $k_{2}^{{\text{c}}_{j}}$ parameters, $I_{4}^{\text{m}}$ is also identical to $I_{4}^{{\text{c}}_{j}}$ defined in Equation (10) (replacing c_*j*_ by m) and $\lambda_{\text{act}}=\lambda^{\text{m}}\lambda_{0}^{\text{m}}$ is the active stretch in the fiber direction, where $\lambda^{\text{m}}=\|Fa_{0}^{\text{m}}\|=\sqrt{C:\left(a_{0}^{\text{m}}⊗a_{0}^{\text{m}}\right)}$.

Moreover, σ_actmax_ denotes the maximal active Cauchy stress and λ_max_ and λ_0_ are parameters denoting the active stretches at maximum and zero active stresses, respectively.

It is worthwhile noting that the same strain energy density functions are assumed all across the aorta. However, different material properties and mass densities of the individual constituents are assigned to each layer (media and adventitia). Note that the intima layer is disregarded in this work, due to its relatively thin thickness.

#### 2.1.4. Stress Evaluation

Having Equation (6), it is now possible to evaluate the second Piola-Kirchhoff stress tensor using the following relations

(12) $$\begin{array}{l} S=2\frac{\partial W}{\partial C}=2\underset{i}{\sum }\varrho_{0}^{i}\frac{\partial W^{i}}{\partial C}=\underset{i}{\sum }\varphi^{i}S^{i} \end{array}$$

where ***S***^*i*^ is the Piola-Kirchhoff stress of constituent *i* and φ^*i*^ its volume fraction in the current configuration, which may be written, such as

(13a) $$\begin{array}{l} \varphi^{e}S^{\text{e}}=\varrho_{0}^{\text{e}}(t)[{\left(J_{\text{el}}^{\text{e}}\right)}^{-2/3}\mu^{\text{e}}\left({\left(C_{\text{gr}}^{\text{e}}\right)}^{-1}-\frac{1}{3}\text{tr}\left(C_{\text{el}}^{\text{e}}\right)C^{-1}\right)\\ \;+\kappa J_{\text{el}}^{\text{e}}(J_{\text{el}}^{\text{e}}-1)C^{-1}]; \end{array}$$

(13b) $$\begin{array}{l} \varphi^{{\text{c}}_{j}}S^{{\text{c}}_{j}}=\varrho_{0}^{{\text{c}}_{j}}\left(t\right)\frac{2\left[k_{1}^{{\text{c}}_{j}}\left(I_{4}^{{\text{c}}_{j}}-1\right)\text{exp}\left(k_{2}^{{\text{c}}_{j}}{\left(I_{4}^{{\text{c}}_{j}}-1\right)}^{2}\right)\right]}{\|F_{gr}^{{\text{c}}_{j}}a_{0}^{{\text{c}}_{j}}{\|}^{2}}a_{0}^{{\text{c}}_{j}}⊗a_{0}^{{\text{c}}_{j}}; \end{array}$$

(13c) $$\begin{array}{l} \varphi^{m}S^{\text{m}}=\varrho_{0}^{\text{m}}\left(t\right)\frac{2\left[k_{1}^{\text{m}}\left(I_{4}^{\text{m}}-1\right)\text{exp}\left(k_{2}^{\text{m}}{\left(I_{4}^{\text{m}}-1\right)}^{2}\right]\right)}{\|F_{gr}^{\text{m}}a_{0}^{\text{m}}{\|}^{2}}a_{0}^{\text{m}}⊗a_{0}^{\text{m}}\\ \begin{array}{l} \;+\frac{\varrho_{0}^{m}\left(t\right)}{\varrho_{0}\left(0\right)}\frac{\sigma_{\text{actmax}}}{\left[C:\left(a_{0}^{\text{m}}⊗a_{0}^{\text{m}}\right)\right]}\left(1-\frac{{\left(\lambda_{\text{max}}-\lambda_{\text{act}}\right)}^{2}}{{\left(\lambda_{\text{max}}-\lambda_{0}\right)}^{2}}\right)a_{0}^{\text{m}}⊗a_{0}^{\text{m}}. \end{array} \end{array}$$

The obtained second Piola-Kirchhoff stress can simply be pushed forward in order to compute the Cauchy stress tensor

(14) $$\begin{array}{l} \sigma^{i}=\frac{1}{J}FS^{i}F. \end{array}$$

Therefore, the Cauchy stress for each individual constituent can be expressed as

(15a) $$\begin{array}{l} \varphi^{e}\sigma^{\text{e}}=\varrho^{\text{e}}\left(t\right){\left(J_{\text{el}}^{\text{e}}\right)}^{-2/3}\mu^{\text{e}}\left(B_{\text{el}}^{\text{e}}-\frac{1}{3}\text{tr}\left(C_{\text{el}}^{\text{e}}\right)I\right)\\ \begin{array}{l} \;+\varrho_{0}^{\text{e}}\left(t\right){\left(J_{\text{gr}}^{\text{e}}\right)}^{-1}\kappa \left(J_{\text{el}}^{\text{e}}-1\right)I; \end{array} \end{array}$$

(15b) $$\begin{array}{l} \varphi^{{\text{c}}_{j}}\sigma^{{\text{c}}_{j}}=2\varrho^{{\text{c}}_{j}}\left(t\right)\left[k_{1}^{{\text{c}}_{j}}\left(I_{4}^{{\text{c}}_{j}}-1\right)\text{exp}\left(k_{2}^{{\text{c}}_{j}}{\left(I_{4}^{{\text{c}}_{j}}-1\right)}^{2}\right)\right]\frac{\left(Fa_{0}^{{\text{c}}_{j}}\right)⊗\left(Fa_{0}^{{\text{c}}_{j}}\right)}{\|F_{gr}^{{\text{c}}_{j}}a_{0}^{{\text{c}}_{j}}{\|}^{2}}; \end{array}$$

(15c) $$\begin{array}{l} \varphi^{m}\sigma^{\text{m}}=2\varrho^{\text{m}}\left(t\right)\left[k_{1}^{\text{m}}\left(I_{4}^{\text{m}}-1\right)\text{exp}\left(k_{2}^{\text{m}}{\left(I_{4}^{\text{m}}-1\right)}^{2}\right]\right)\frac{\left(Fa_{0}^{\text{m}}\right)⊗\left(Fa_{0}^{\text{m}}\right)}{\|F_{gr}^{\text{m}}a_{0}^{\text{m}}{\|}^{2}}\\ \begin{array}{l} \;+\frac{\varrho^{m}\left(t\right)}{\varrho_{0}\left(0\right)}\sigma_{\text{actmax}}\left(1-\frac{{\left(\lambda_{\text{max}}-\lambda^{\text{m}}\lambda_{0}^{\text{m}}\right)}^{2}}{{\left(\lambda_{\text{max}}-\lambda_{0}\right)}^{2}}\right)\frac{\left(Fa_{0}^{\text{m}}\right)⊗\left(Fa_{0}^{\text{m}}\right)}{\|Fa_{0}^{\text{m}}{\|}^{2}}. \end{array} \end{array}$$

where $B_{\text{el}}^{\text{e}}=F_{\text{el}}^{\text{e}}{F_{\text{el}}^{\text{e}}}^{T}$ is the elastic part of the left Cauchy-Green stretch tensor for elastin.

In Equation (15c), λ_act_ was replaced by $\lambda^{\text{m}}\lambda_{0}^{\text{m}}$, which means that we assume there is a continuous increase of the active stretch of SMCs during ATAA progression (${\overset{∙}{\lambda }}^{\text{m}}>0$). This is a different assumption of the one made by Braeu et al. (2017) who assumed fast remodeling of SMCs, which implied that SMCs were able to keep the same active stretch throughout the G&R process (λ_act_ = 1 meaning that ${\hat{\lambda }}_{\text{act}}=\lambda^{\text{m}}$). In the current work, we want to investigate computationally the opposite situation, where SMC would proliferate at an extremely low rate (Owens et al., 2004), which implies that the active stretch has to accommodate ATAA expansion.

#### 2.1.5. Growth and Remodeling

In this work, a two-layer arterial model was considered where the rate of mass degradation or deposition of collagen in both layers was computed, such as

(16a) $$\begin{array}{l} {\dot{\varrho }}_{0}^{{\text{c}}_{j}}\left(t\right)=\varrho_{0}^{{\text{c}}_{j}}\left(t\right)k_{\sigma }^{{\text{c}}_{j}}\frac{\sigma^{\text{m}}-\sigma_{\text{h}}^{\text{m}}}{\sigma_{\text{h}}^{\text{m}}}+{\dot{D}}_{\text{g}}^{{\text{c}}_{j}}\text{ in the media}, \end{array}$$

(16b) $$\begin{array}{l} {\dot{\varrho }}_{0}^{{\text{c}}_{j}}\left(t\right)=\varrho_{0}^{{\text{c}}_{j}}\left(t\right)k_{\sigma }^{{\text{c}}_{j}}\frac{\sigma^{{\text{c}}_{j}}-\sigma_{\text{h}}^{{\text{c}}_{j}}}{\sigma_{\text{h}}^{{\text{c}}_{j}}}+{\dot{D}}_{\text{g}}^{{\text{c}}_{j}}\text{ in the adventitia}, \end{array}$$

where $k_{\sigma }^{{\text{c}}_{j}}$ stands for (typically constant) collagen growth parameter, $\sigma_{\text{h}}^{\text{m}}$ and $\sigma_{\text{h}}^{{\text{c}}_{j}}$ denote the average SMCs and collagen fiber stresses at homeostasis, σ^m^ and $\sigma^{{\text{c}}_{j}}$ are the current stress of the extant SMCs and collagen fibers, respectively. Moreover, it is assumed that elastin can be only subjected to degradation if its mass loss cannot be compensated by new elastin deposition (${\dot{\varrho }}_{0}^{\text{e}}\left(t\right)={\dot{D}}_{\text{g}}^{\text{e}}$). In this case, ${\dot{D}}_{\text{g}}^{\text{e}}$ is the so-called generic function of the local elastin degradation rate, defined as

(17) $$\begin{array}{l} {\dot{D}}_{\text{g}}^{\text{e}}\left(X,t\right)=-\frac{\varrho_{0}^{\text{e}}\left(X,t\right)}{T^{\text{e}}}-\frac{D_{\text{max}}}{t_{\text{dam}}}\varrho_{0}^{\text{e}}\left(X,0\right)e^{-\frac{t}{t_{\text{dam}}}}, \end{array}$$

whose objective is to describe additional mass deposition or mass degradation (e.g., as a result of any damage) in elastin. *D*_max_ is the maximum damage length, ***X*** is the material position, *L*_dam_ and *t*_dam_ are the spatial and the temporal damage spread parameters, respectively, and *T*^e^ is the average turnover time for elastin constituent.

Having mass turnover relationships, it is now possible to capture the inelastic deformation induced by G&R. The evolution of the inelastic remodeling deformation gradient of constituent *i* at time *t* can be evaluated by solving the following system of equations (Cyron et al., 2016; Braeu et al., 2017)

(18) $$\begin{array}{l} \left[\frac{{\dot{\varrho }}_{0}^{i}\left(t\right)}{\varrho_{0}^{i}\left(t\right)}+\frac{1}{T^{i}}\right]\left[S^{i}-S_{\text{pre}}^{i}\right]={\left[2\frac{\partial S_{i}}{\partial C_{\text{el}}^{i}}:\left(C_{\text{el}}^{i}L_{\text{r}}^{i}\right)\right]}_{F_{\text{mix}},F_{\text{g}}^{i}}, \end{array}$$

where ***S*** is the second Piola-Kirchhoff stress tensor, $L_{\text{r}}^{i}={\dot{F}}_{\text{r}}^{i}{F_{\text{r}}^{i}}^{-1}$ is the remodeling velocity gradient, *T*^*i*^ is the period within which a mass increment is degraded and replaced by a new mass increment, known as the average mass turnover time. It is worthwhile noting that $S_{\text{pre}}^{i}$ denotes the deposition pre-stress, the net mass production rate is already defined in Equation (16). In the case of elastin, it is assumed that this constituent is not produced any longer during adulthood and it undergoes a slow degradation with a half-life time of several decades (Cyron and Humphrey, 2016; Braeu et al., 2017). Consequently, the remodeling velocity gradient and, subsequently, the remodeling deformation gradient are zero ($L_{\text{r}}^{\text{e}}=F_{\text{r}}^{\text{e}}=0$). Hence, elastin growth can essentially be computed on the basis of its degradation rate ${\dot{D}}_{\text{g}}^{\text{e}}$, defined at Equation (17).

The inelastic growth deformation gradient is derived by summing the growth-related deformation gradient rates of each individual constituent, such as

(19) $$\begin{array}{l} {\dot{F}}_{\text{g}}^{i}={\dot{F}}_{\text{g}}=\overset{n}{\underset{i=1}{\sum }}\frac{{\dot{\varrho }}_{0}^{i}\left(t\right)}{\varrho_{0}\left(t\right)\left[{F_{\text{g}}^{i}}^{\text{-T}}:a_{\text{g}}^{i}⊗a_{\text{g}}^{i}\right]}a_{\text{g}}^{i}⊗a_{\text{g}}^{i}, \end{array}$$

where $a_{\text{g}}^{i}$ is a unit vector along the growth direction per individual constituents which, for instance, can represent an anisotropic growth across the direction of the arterial wall thickness and $\varrho_{0}\left(t\right)=\underset{i}{\sum }\varrho_{0}^{i}\left(t\right)$.

### 2.2. Numerical Implementation

A three-dimensional (3D) structural mesh, made of hexahedral elements, is reconstructed across the arterial wall using the Abaqus finite-element package (Hibbit et al., 2011). Each element has a regionally different mass density and contains a mixture of elastin, collagen fiber families and SMCs. The structural mesh implies the edge of each element is locally aligned with the material (i.e., radial, circumferential, and axial) directions of the arterial wall. In the case of non-perfectly cylindrical geometries, the radial direction is defined as the outward normal direction to the luminal surface and the axial direction is considered parallel to the luminal centerline along the blood flow stream. Finally, the circumferential direction is defined perpendicularly to the other two introduced directions.

The proposed model is implemented in Abaqus, through a coupled User-defined MATerial subroutine (UMAT) (Hibbit et al., 2011). The evolution of the arterial wall deformation is obtained by approximating the solution of the non-linear system of equations using the FEM approach, incremented through the Newton-Raphson method. Each time step of the simulation represents 1 month in the real (physical) timescale. The G&R deformation gradients are obtained at each time step on the basis of the stresses assessed at the previous step. It is worthwhile noting that the initial time step is only assumed to satisfy homeostatic conditions and the next time step is triggered by arterial dilatation through the G&R progression.

### 2.3. Description of the Case Study

**Figure 1:**
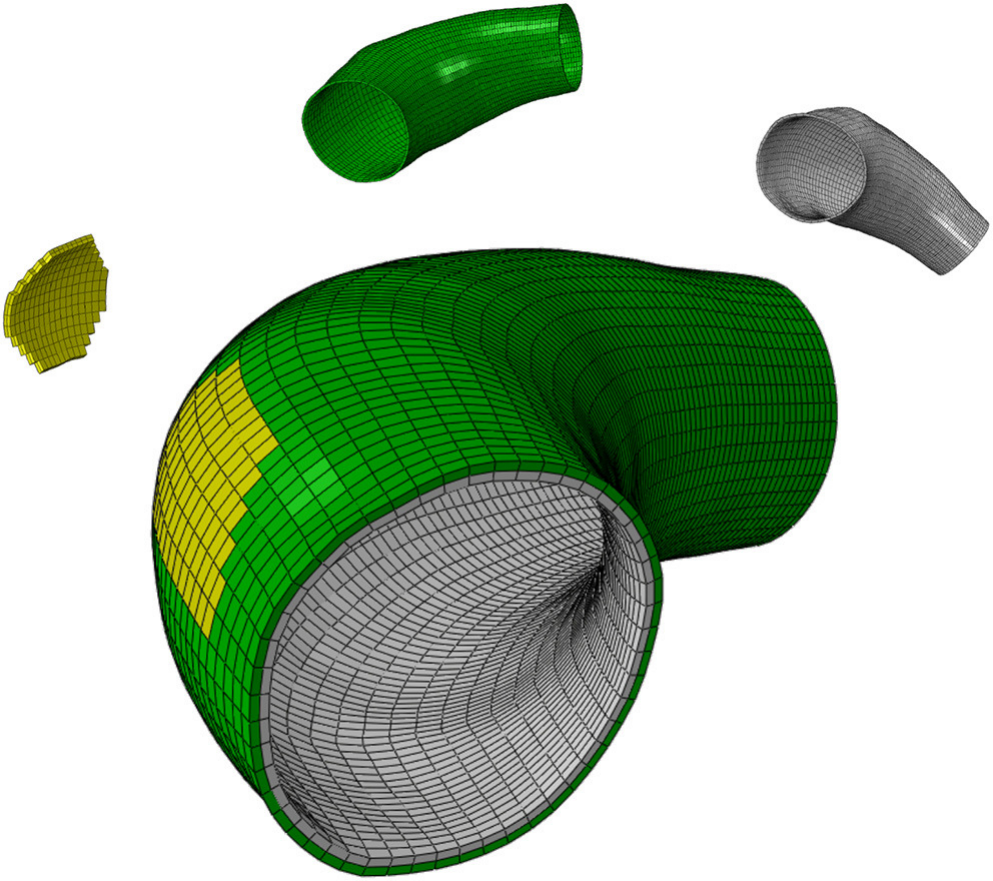
The computational domain of the ATAA geometry composed of the localized elastin degradation region, media layer and adventitia layer shown in yellow, gray, and green colors, respectively.

Here, the model of Mousavi et al. (2019) is applied on a real human ATAA geometry in order to predict G&R of a patient-specific arterial wall. To do so, the preoperative Computerized Tomography (CT) scan of the patient as obtained after informed consent from a donor undergoing elective surgery for ATAA repair at CHU-SE (Saint-Etienne, France). Although the lumen of the aneurysm was clearly visible in the Digital Imaging and COMmunications (DICOM) file, it was not trivial to detect the aneurysm surface. A non-automatic segmentation of the CT image slices was performed using MIMICS (v. 10.01, Materialize NV) to reconstruct the ATAA geometry. The obtained geometry was assigned as the reference configuration and was structurally meshed with 7,700 hexahedral elements. A wall thickness of 2.38 mm was defined evenly in the reference configuration, yielding an average thickness of 2.67 mm at zero pressure state as the measured thickness in the supplied sample (Farzaneh et al., 2019). Figure 1 shows the computational domain in which the localized elastin degradation region is illustrated along with the media and adventitia layers.

The material parameters (Table 1), such as deposition stretches of collagen and other material parameters are the ones reported in references (Bellini et al., 2014; Braeu et al., 2017) or result from previous calibration against experimental data (Davis et al., 2016). Note that 97% of total elastin, 100% of total SMC, and 15% of total axial and diagonal collagen fibers are assigned to the media layer. Conversely, 3% of total elastin, 85% of total axial and diagonal collagen, and 100% of total circumferential collagen are assigned to the adventitia layer (Bellini et al., 2014; Mousavi and Avril, 2017; Mousavi et al., 2018).

**Table 1:** Material parameters employed for two-layer patient-specific human ATAA model adapted from Mousavi et al. (2019).

**Parameters**	**Values**	**Units**
$\alpha^{c_{j}},j=1,2,3,4$	$\left\{0,\frac{\pi }{2},\pm \frac{\pi }{4}\right\}$	[Rad]
μ^e^	82	[J/kg]
κ	100μ^e^	[J/kg]
$k_{1}^{{\text{c}}_{j},\text{c}}=k_{1}^{\text{m},\text{c}}$	15	[J/kg]
$k_{2}^{{\text{c}}_{j},\text{c}}=k_{2}^{\text{m},\text{c}}$	1.0	[–]
$k_{1}^{{\text{c}}_{j},\text{t}}$	105	[J/kg]
$k_{2}^{{\text{c}}_{j},\text{t}}$	0.13	[–]
$k_{1}^{\text{m},\text{t}}$	10	[J/kg]
$k_{2}^{\text{m},\text{t}}$	0.1	[–]
$\varrho_{0}^{\text{e}}$	250	[kg/m^3^]
$\varrho_{0}^{{\text{c}}_{j}}$	460	[kg/m^3^]
$\varrho_{0}^{\text{m}}$	280	[kg/m^3^]
${\lambda_{0}^{\text{e}}}_{z}$	1.3	[–]
${\lambda_{0}^{\text{c}}}_{j}$	1.1	[–]
$\lambda_{0}^{\text{m}}$	1.1	[–]
λ_0_	0.8	[–]
λ_max_	{1, 1.1, 1.4}	[–]
σ_a*ctmax*_	54	[kPa]
*T*^e^	101	[Years]
$T^{{\text{c}}_{j}}$	101	[Days]
*T*^m^	101	[Days]
*t*_dam_	{20, 40, 80}	[Days]
*D*_max_	0.5	[–]

*$\alpha^{c_{1}}$, $\alpha^{c_{2}}$, $\alpha^{c_{3}}$, and $\alpha^{c_{4}}$ are axial, circumferential, and two diagonal directions of collagen fiber families, respectively*.

The geometry is subjected to a luminal pressure of 80 mmHg (diastole), an axial deposition stretch of ${\lambda_{0}^{\text{e}}}_{z}=1.3$ defined for elastin while the deposition stretches of collagen families and SMCs are set to $\lambda_{0}^{{\text{c}}_{j}}=\lambda_{0}^{\text{m}}=1.1$. The spatially varying circumferential deposition stretch of elastin is determined to ensure equilibrium with the luminal pressure using the iterative algorithm presented in Mousavi and Avril (2017). The applied boundary conditions to the geometry are defined as fixed at ends of the ATAA model in axial and circumferential directions (*z* and θ) while free (no-traction) boundary condition are assigned in the radial direction (*r*).

Guzzardi et al. (2015) found that regions of the aortic wall subjected to the localized effects of the jet flow expelled from the heart underwent greater elastin degradation associated with localized vessel wall remodeling. Therefore, and on the basis of these findings, a localized elastin degradation is considered here and its G&R effects are numerically simulated on the patient-specific ATAA geometry. The localized region of elastin degradation is shown in Figure 1.

### 2.4. Sensitivity Analysis

A parametric study was carried out about the effects of three parameters on G&R progression:

- the time at which the artery is maximally damaged (*t*_dam_),
- the rate of collagen deposition ($k_{\sigma }^{{\text{c}}_{j}}/T^{{\text{c}}_{j}}$),
- the maximum contractility of SMCs (λ_max_).

The different values for *t*_dam_ follow the assumption that patient-specific G&R strongly relates to the temporal and spatial distributions of elastin degradation (Watton et al., 2004; Bellini et al., 2014; Braeu et al., 2017). Different values of $k_{\sigma }^{{\text{c}}_{j}}/T^{{\text{c}}_{j}}$ were considered since the rate of collagen deposition determines how fast the tissue adapts to elastin loss (Mousavi et al., 2019). As the focus of this work is on the active contribution of SMCs on G&R, different values were also considered for λ_max_, possibly corresponding to possibly pathologic variations of intracellular filament structure in SMCs (Liu et al., 2008; Mantella et al., 2015).

## 3. Results

### 3.1. SMCs Active Stress Contribution

In order to quantify the contribution of SMCs active tone in the stress distribution field, two ratios were considered, namely (1) the ratio of the SMCs active stress with respect to the SMCs (active and passive) stresses (*R*^m^); and (2) the ratio between the SMCs active stress and the total stress field (*R*^t^).

(20) $$\begin{array}{l} R^{\text{m}}=\frac{\sigma_{\text{act}}^{\text{m}}}{\sigma^{\text{m}}};\;R^{\text{t}}=\frac{\sigma_{\text{act}}^{\text{m}}}{\sigma^{\text{t}}}. \end{array}$$

**Figure 2:**
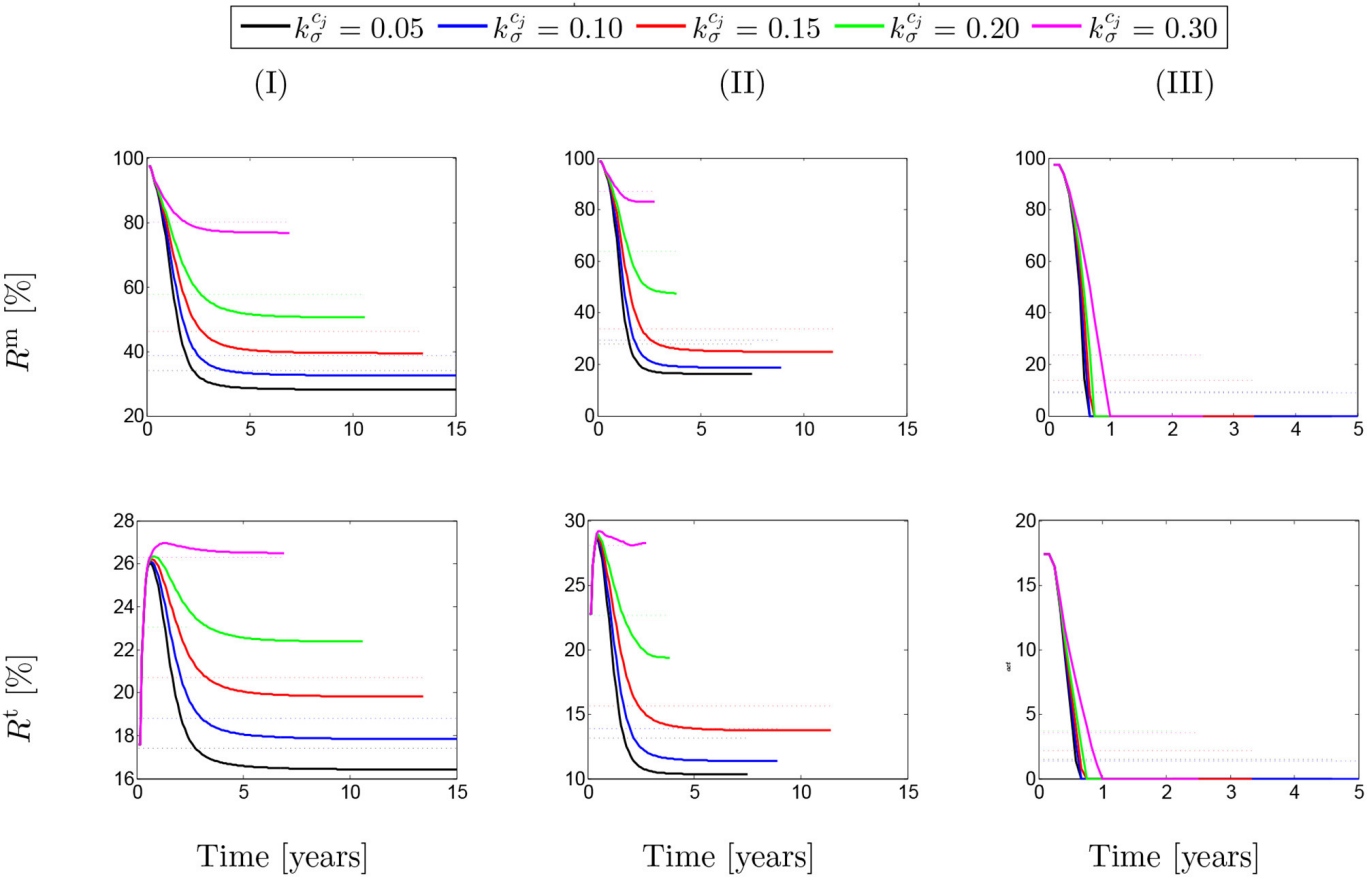
The evolution of the stress ratios {*R*^m^, *R*^t^} with respect to physical time *t*. The dotted lines denote the average value for each color line correspondingly. The results obtained for a two-layer thick-wall patient-specific human ATAA responding to a regional elastin degradation for 15 years. Different rates of collagen deposition $k_{\sigma }^{{\text{c}}_{j}}/T^{{\text{c}}_{j}}$ are considered depending on the (column-wise presentation of the) maximum SMCs contractility (I) λ_max_ = 1.4; (II) λ_max_ = 1.1; and (III) λ_max_ = 1.0 for a fixed value of *t*_dam_ = 40.

**Figure 3:**
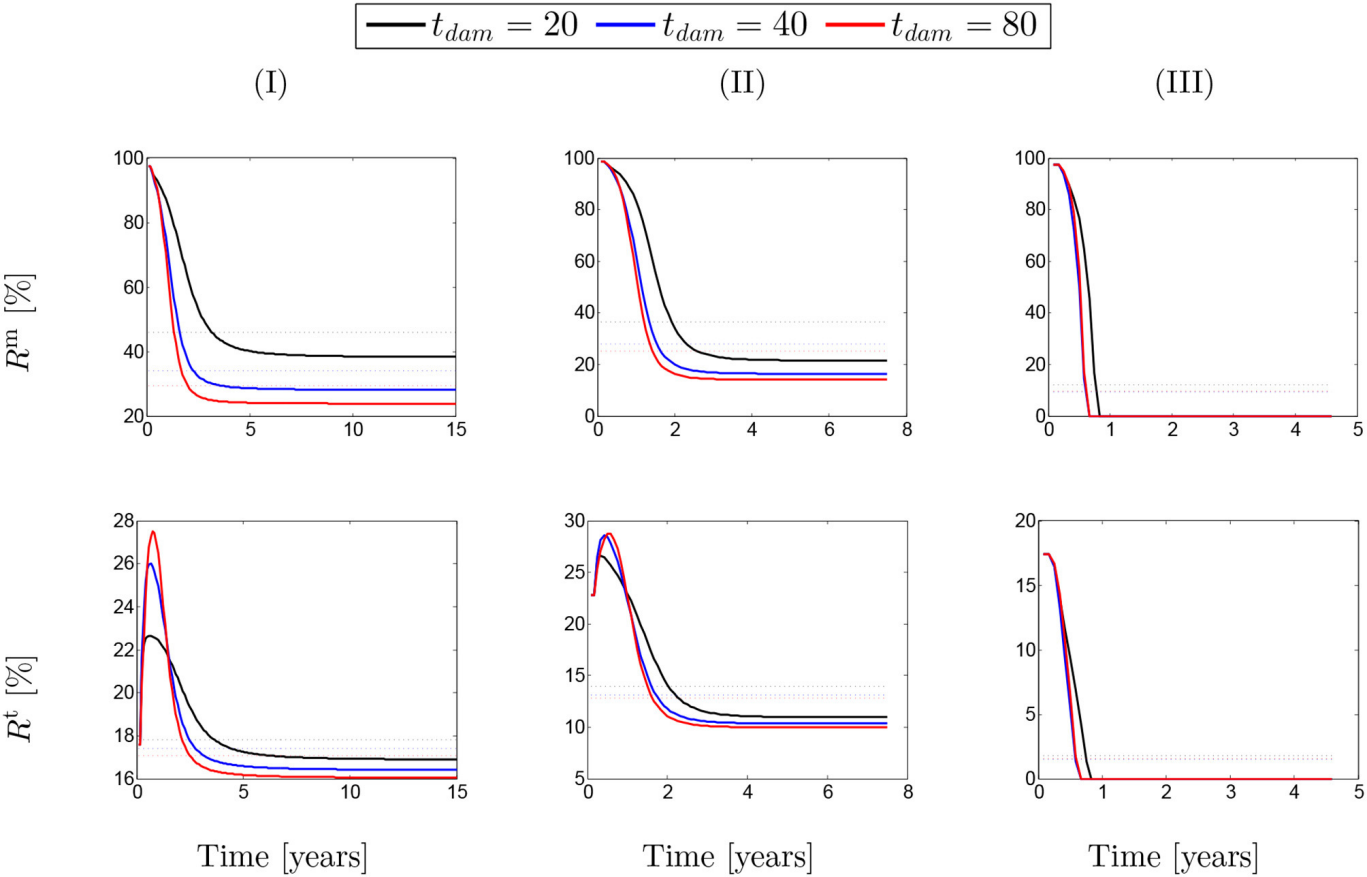
The evolution of the stress ratios {*R*^m^, *R*^t^} with respect to physical time *t*. The dotted lines denote the average value for each color line correspondingly. The results obtained for a two-layer thick-wall patient-specific human ATAA responding to a regional elastin degradation for 15 years. Different damage times *t*_dam_ are considered depending on the (column-wise presentation of the) maximum SMCs contractility (I) λ_max_ = 1.4; (II) λ_max_ = 1.1; and (III) λ_max_ = 1.0 for a fixed value of $k_{\sigma }^{{\text{c}}_{j}}=0.05$.

These ratios are shown in percentage depending on the maximum SMCs active stretches for different rates of collagen deposition (Figure 2) and damage times (Figure 3) due to elastin degradation.

Figure 2 shows that these ratios are different depending on the amount of collagen deposition. The active stress includes an average value (dotted-lines in Figure 2) between 25 and 85% of the total SMCs stresses. However, this average value has reduced to an approximate value of *R*^m^ = 20% in the case of λ_max_ = 1.0 when the SMCs lose their functionality. This contribution of active stress with respect to the total stress field of the mixture is the average value between 14 and 28%. Indeed, the SMCs contribution to the total stress field has reduced down to *R*^t^ = 4% in the critical condition of λ_max_ = 1.0. Notice here that, in general, the solution has computationally converged faster by increasing the rate of collagen deposition as the artery becomes thicker and less displacement is expected. The convergence criteria state that the average value of the artery dilation should not exceed 10^−5^m with respect to the previous time step and reads

(21) $$\begin{array}{l} {\left(u_{\text{Ave}}\right)}^{n}-{\left(u_{\text{Ave}}\right)}^{n-1}<0.01;\;u_{\text{Ave}}=100\times \frac{\sum_{i=1}^{N_{e}}\|u_{i}\|}{N_{e}\times T}, \end{array}$$

where ***u***_Ave_ is the average displacement of all mesh elements of the artery, $\|u_{i}\|=\sqrt{u_{1}^{2}+u_{2}^{2}+u_{3}^{2}}$ is the norm of elemental displacement, *n* stands for the time step, *T* = 2.5 × 10^−3^ denotes the thickness of the artery and *N*_*e*_ is the number of Hexahedral elements.

A similar behavior of SMCs active contribution can be observed in Figure 3 for different values of damage time *t*_dam_. In this case, the active stress has averagely represented between 25 and 45% of SMCs stresses and between 13 and 17% of the total stress. This has reduced to <10% in the critical condition of λ_max_ = 1.0.

### 3.2. SMCs Stress-Stretch Curve

**Figure 4:**
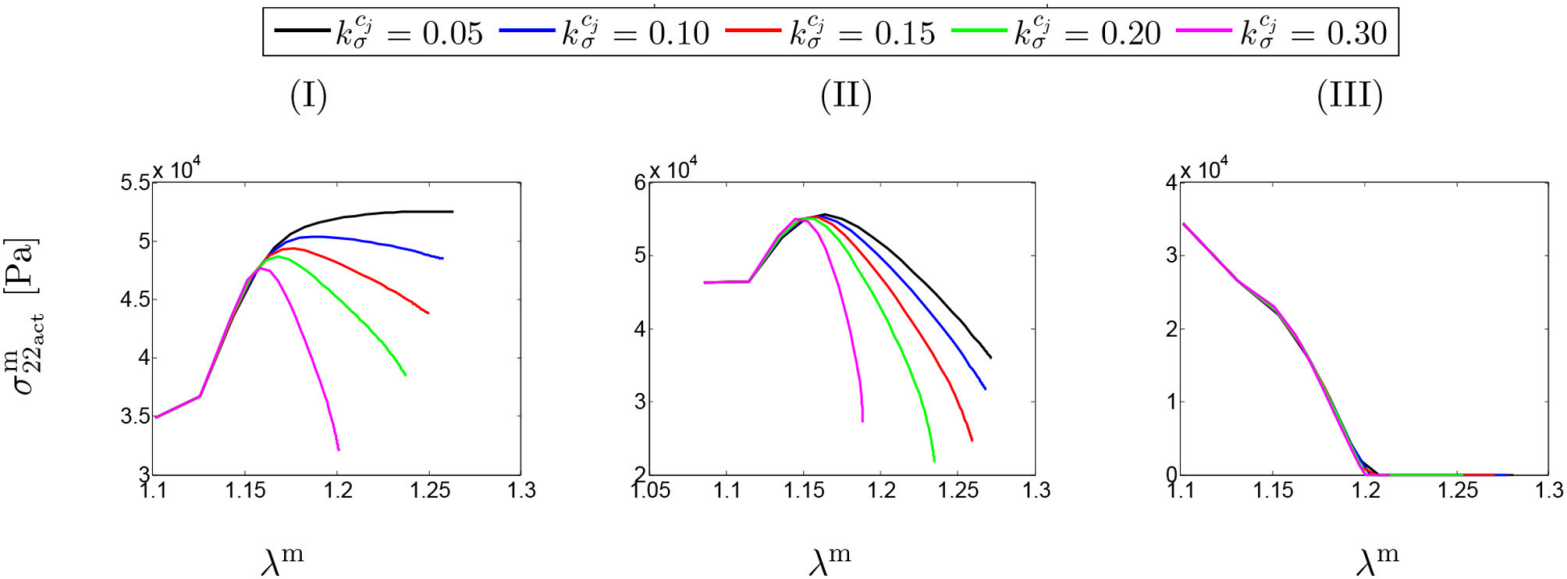
SMCs stress-stretch ($\sigma_{22_{\text{act}}}^{\text{m}}$–λ^m^) curve: the results obtained for a two-layer thick-wall patient-specific human ATAA responding to a regional elastin degradation for 15 years. Different rates of collagen deposition $k_{\sigma }^{{\text{c}}_{j}}/T^{{\text{c}}_{j}}$ are considered depending on the (column-wise presentation of the) maximum SMCs contractility **(I)** λ_max_ = 1.4; **(II)** λ_max_ = 1.1; and **(III)** λ_max_ = 1.0 for a fixed value of *t*_dam_ = 40 [days].

Figure 4 shows SMCs stress-stretch curves. In fact, the SMCs active stress in the circumferential direction is plotted vs. the SMCs stretches for different values of maximum SMCs contractility (λ_max_). The curves are comparatively depicted in Figure 4A for various speeds of collagen deposition ($k_{\sigma }^{{\text{c}}_{j}}/T^{{\text{c}}_{j}}$) when the maximum active stretch is larger than the initial deposition stretch ($\lambda_{\text{max}}>\lambda_{0}^{\text{m}}$). It can be observed that the active stress is initially increased while the artery is stretched due to G&R. This monotonically increasing trend remains up to the point in which SMCs reach their maximum contractility and, then, as expected from their Hill functional behavior, it begins to decrease depending on the amount of collagen deposition.

Figure 4B illustrates the stress-stretch curve for various rates of collagen deposition ($k_{\sigma }^{{\text{c}}_{j}}/T^{{\text{c}}_{j}}$) when the maximum active stretch is larger than the initial deposition stretch ($\lambda_{\text{max}}≃\lambda_{0}^{\text{m}}$). The above-mentioned explanations regarding SMCs behavior still hold by noting that the stress has almost reached its maximum value for all values of $k_{\sigma }^{{\text{c}}_{j}}$. Moreover, the decrease in maximum contractility (λ_max_) has led to a larger decrease in active stress in comparison to the previous case, once reaching the maximum active stress value.

Interestingly, the active stress even drops and reaches zero (see Figure 4III). In this case, SMCs may fail to adhere onto and pull the extracellular matrix and eventually go into apoptosis. This critical situation happens no matter what rate of collagen deposition is applied.

**Figure 5:**
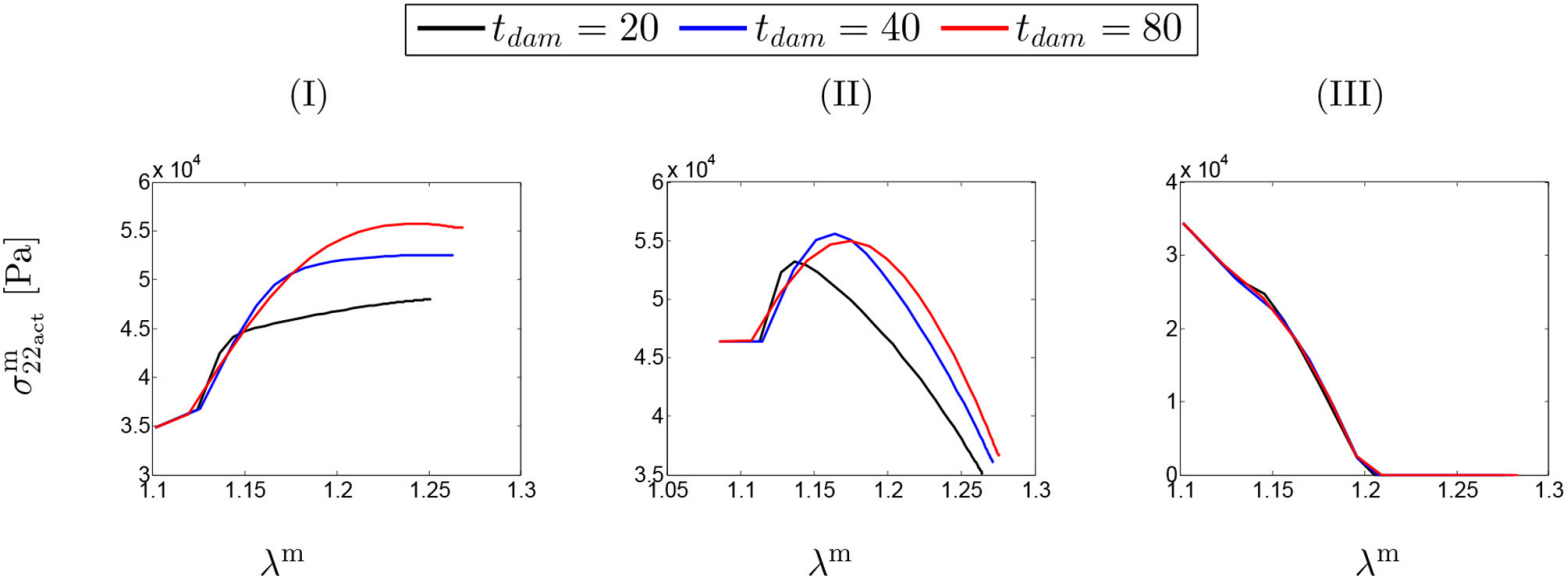
SMCs stress-stretch ($\sigma_{22_{\text{act}}}^{\text{m}}$–λ^m^) curve: the results obtained for a two-layer thick-wall patient-specific human ATAA responding to a regional elastin degradation for 15 years. Different damage times *t*_dam_ [days] are considered depending on the (column-wise presentation of the) maximum SMCs contractility **(I)** λ_max_ = 1.4; **(II)** λ_max_ = 1.1; and **(III)** λ_max_ = 1.0 for a fixed value of $k_{\sigma }^{{\text{c}}_{j}}=0.05$.

Figure 5 depicts the SMCs stress-stretch curves compared for different values of maximum SMCs contractility (λ_max_) and plotted for various damage times *t*_dam_. As clearly shown in Figure 5I, the larger damage time has led to more active stress since the artery is allowed to be more dilated, and then develop more stress, before it is damaged due to elastin degradation. In this case in which the maximum active stretch is larger than the initial deposition stretch, the active stress has an increasing trend from the beginning and all curves reach their corresponding maximum stresses and retain it. This behavior can still be related to the inverted parabola shape in Figure 5II when the maximum SMCs contractility is equivalent to the initial deposition stretch. This is evident as the first step (homeostasis) shows no change in developed stress (horizontal line). As shown in Figure 5III, the critical condition of a zero active stress is reached again after some time of G&R.

**Figure 6:**
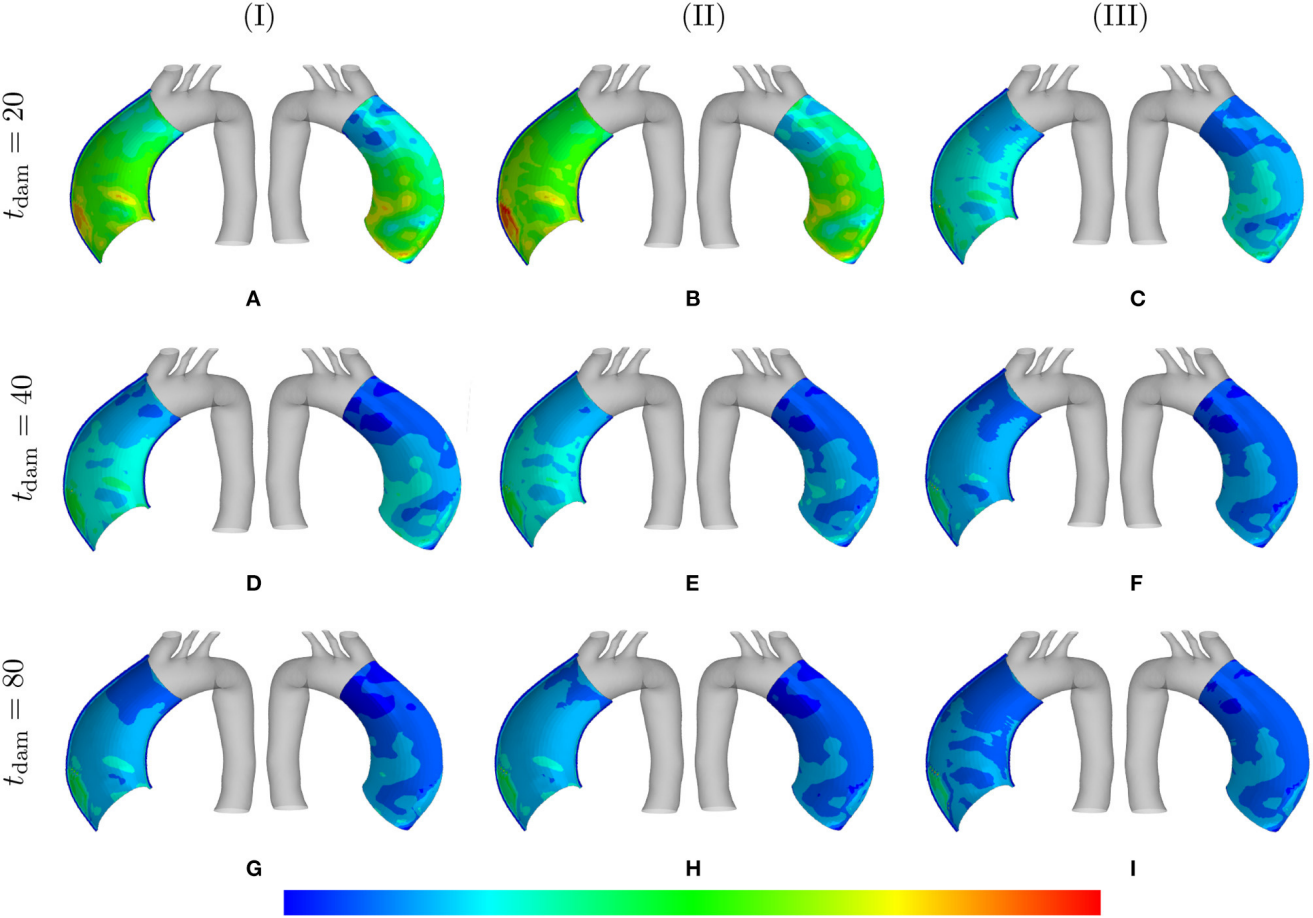
Principal stresses: the stress distributions are comparatively shown at the final physical time (180 months). The results obtained for a two-layer thick-wall patient-specific human ATAA responding to a regional elastin degradation for 15 years. Different damage times *t*_dam_ [days] are considered depending on the (column-wise presentation of the) maximum SMCs contractility **(I)** λ_max_ = 1.4; **(II)** λ_max_ = 1.1; and **(III)** λ_max_ = 1.0 for a fixed value of $k_{\sigma }^{{\text{c}}_{j}}=0.05$. Each column shows the stress distributions, from left to right, for the interior of the arterial wall (cut in half) and for the media layer, respectively. The range of principal stress distributions are **(A)** [6.4e4–3.3e5]; **(B)** [7.1e4– 3.6e5]; **(C)** [7.2e4–6.3e5]; **(D)** [8.2e4–6.2e5]; **(E)** [7.3e4–7.4e5]; **(F)** [7.4e4–8.8e5]; **(G)** [1.1e5–8.1e5]; **(H)** [7.3e4–8.9e5]; **(I)** [8.0e4–1.0e6] in [Pa].

Figure 6 shows the distribution of the first principal stress distribution in the arterial wall for different values of SMC maximum contractility and damage time. The enlargement of the stress range in the artery is evident as the maximum SMCs stretches are reduced. Increasing the damage time results in increasing the stress values as well, with maximum values reached in the region of local elastin degradation.

**Figure 7:**
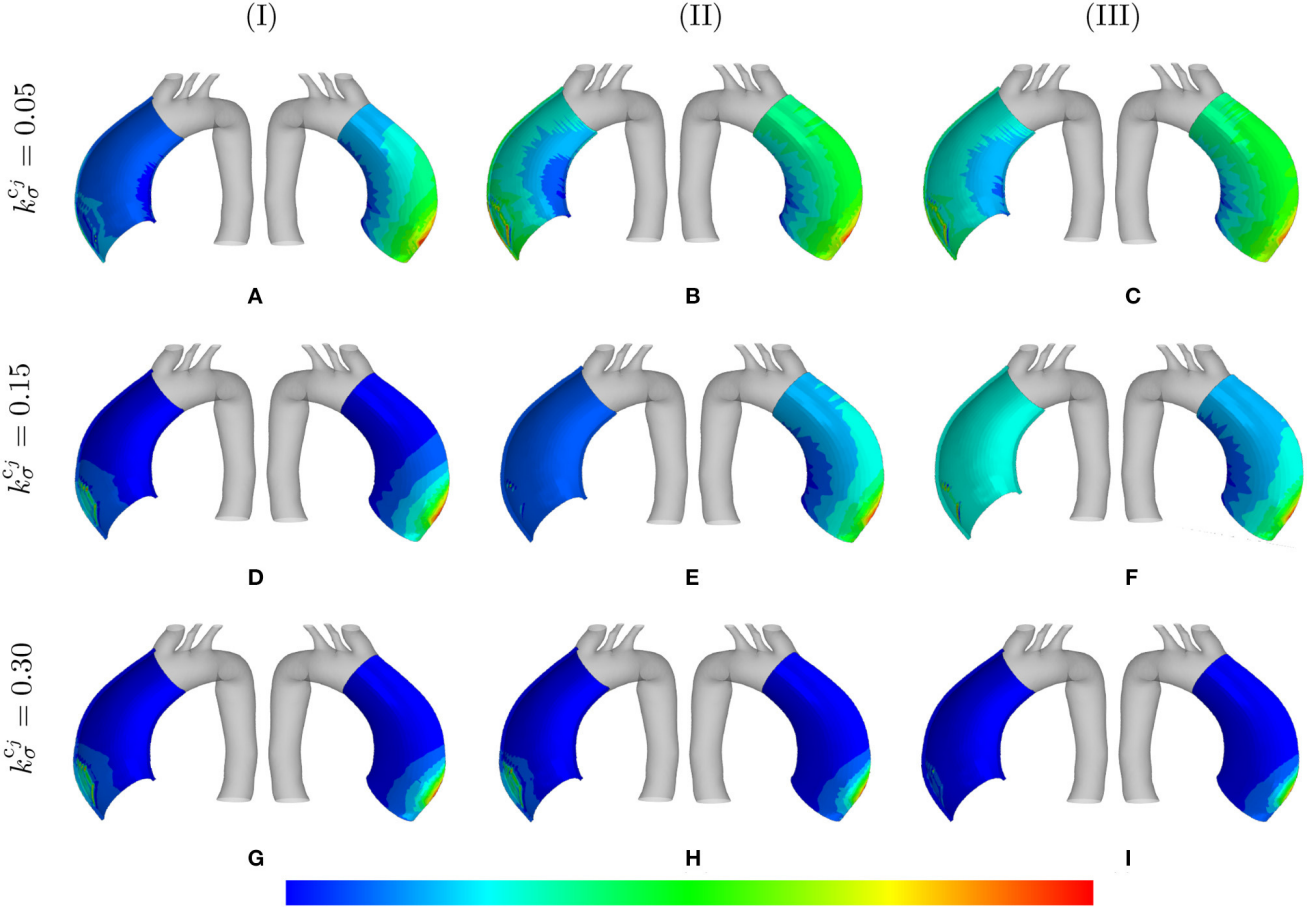
Normalized collagen density: the mass distributions are comparatively shown at the final physical time (180 months). The results obtained for a two-layer thick-wall patient-specific human ATAA responding to a regional elastin degradation for 15 years. Different rates of collagen deposition $k_{\sigma }^{{\text{c}}_{j}}/T^{{\text{c}}_{j}}$ are considered depending on the (column-wise presentation of the) maximum SMCs contractility **(I)** λ_max_ = 1.4; **(II)** λ_max_ = 1.1; and **(III)** λ_max_ = 1.0 for a fixed value of *t*_dam_ = 40 [days]. Each column shows the stress distributions, from left to right, for the interior of the arterial wall (cut in half) and for the adventitia layer, respectively. The range of normalized collagen density distributions are **(A)** [0.95–1.6]; **(B)** [0.99–1.8]; **(C)** [1.0–1.8]; **(D)** [0.99–1.0]; **(E)** [1.0–1.9]; **(F)** [1.1–4.5]; **(G)** [1.1–4.2]; **(H)** [1.5–4.3]; **(I)** [5.1–3.2].

The distribution of the normalized collagen density (with respect to the arterial wall thickness) is depicted in Figure 7 for different values of λ_max_ and $k_{\sigma }^{{\text{c}}_{j}}$.

### 3.3. Stress Evolution

**Figure 8:**
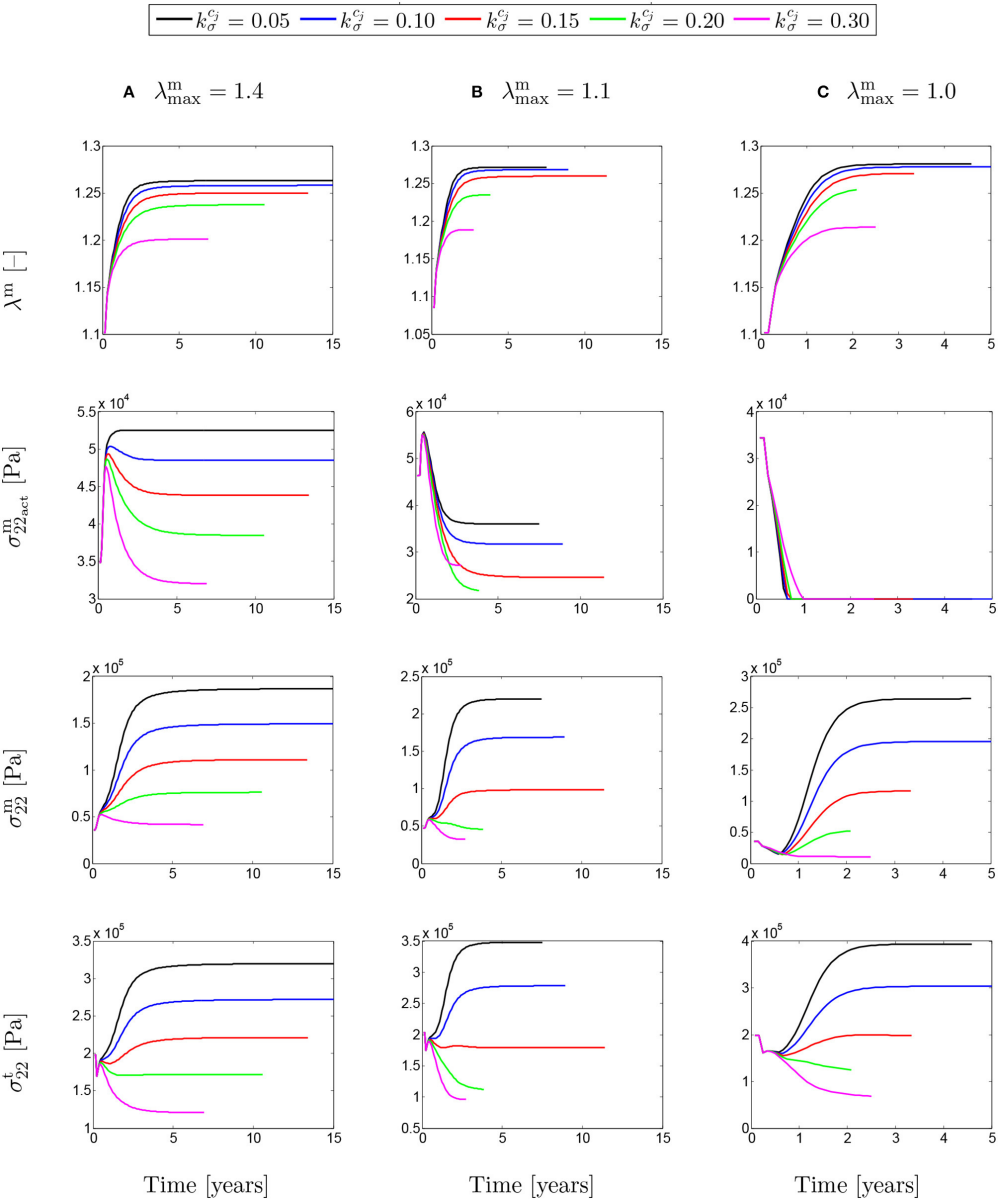
The evolution of the SMCs stretch and stress fields with respect to physical time *t*. The results obtained for a two-layer thick-wall patient-specific human ATAA responding to a regional elastin degradation for 15 years. Different rates of collagen deposition $k_{\sigma }^{{\text{c}}_{j}}/T^{{\text{c}}_{j}}$ are considered depending on the (column-wise presentation of the) maximum SMCs contractility **(A)** λ_max_ = 1.4; **(B)** λ_max_ = 1.1; and **(C)** λ_max_ = 1.0 for a fixed value of *t*_dam_ = 40 [days].

Figure 8 shows the evolution of stretches, active stresses and total stresses of SMCs along with the total stress field of the mixture. The solutions are compared for different values of maximum SMCs active stretches and for different rates of collagen deposition, all for a fixed value of *t*_dam_ = 40. The stretch evolution shows that the ATAA dilation evolves in the opposite direction of the rate of collagen deposition. This occurs as more collagen is deposited for compensating elastin degradation: the thicker the artery and the smaller the displacement. This can be related to the evolution of the active stresses in which the inverted parabola behavior is more evident for the bigger value of $k_{\sigma }^{{\text{c}}_{j}}$. In fact, when deposited collagen has a larger contribution to the stress field, SMCs stresses reach a maximum and, then, decrease afterwards.

**Figure 9:**
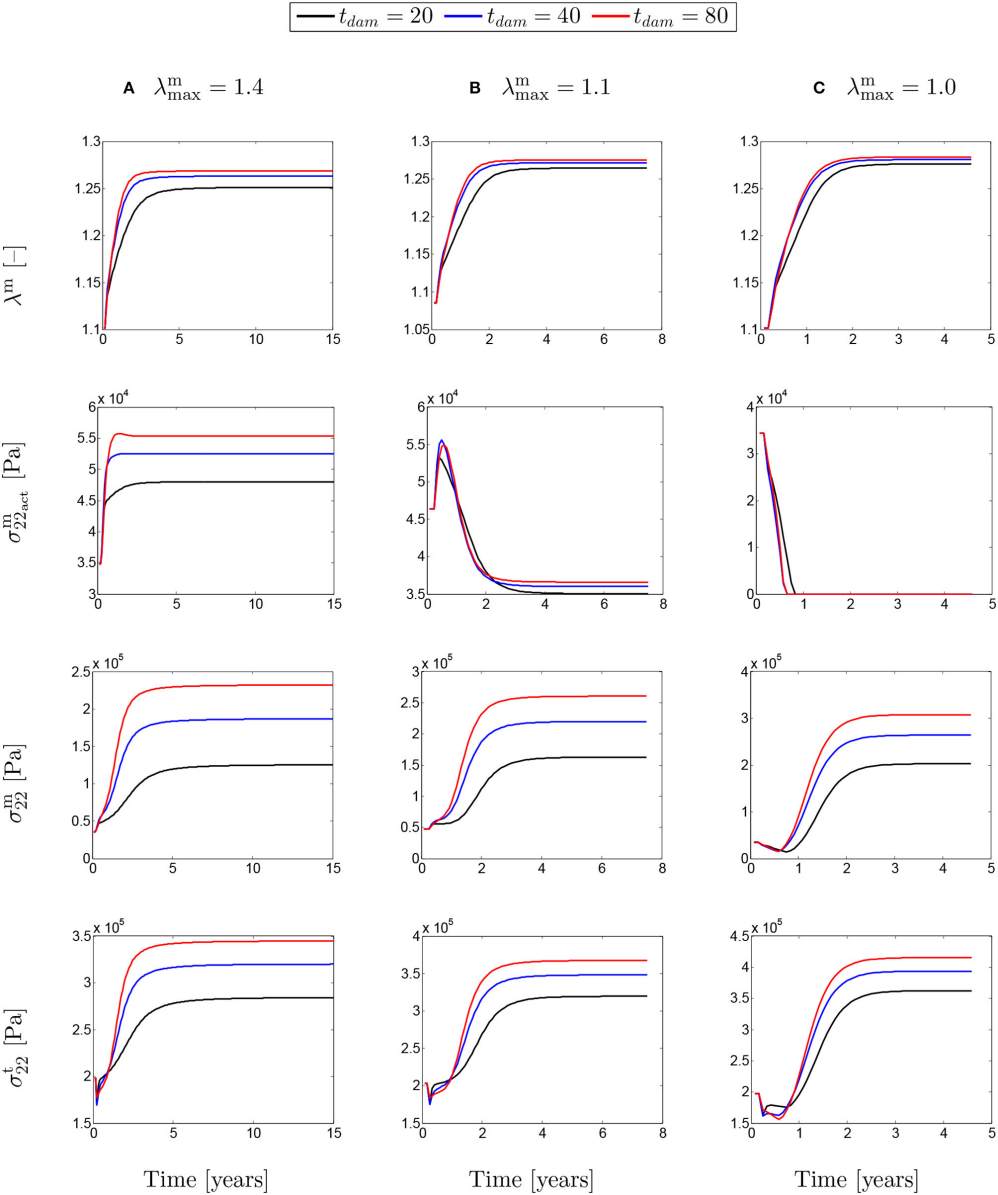
The evolution of the SMCs stretch and stress fields with respect to physical time *t* for a column-wise presented values of **(A)** λ_max_ = 1.4; **(B)** λ_max_ = 1.1; and **(C)** λ_max_ = 1.0. The results obtained for a two-layer thick-wall patient-specific human ATAA responding to a regional elastin degradation for 15 years. Different damage times *t*_dam_ [days] are considered depending on the (column-wise presentation of the) maximum SMCs contractility λ_max_ for a fixed value of $k_{\sigma }^{{\text{c}}_{j}}=0.05$.

The evolutions shown in Figure 9 are similar to the ones shown in Figure 8, but this time for different values of damage time *t*_dam_. In this case, increasing the damage time increased the developed stretch due to G&R in the artery.

**Figure 10:**
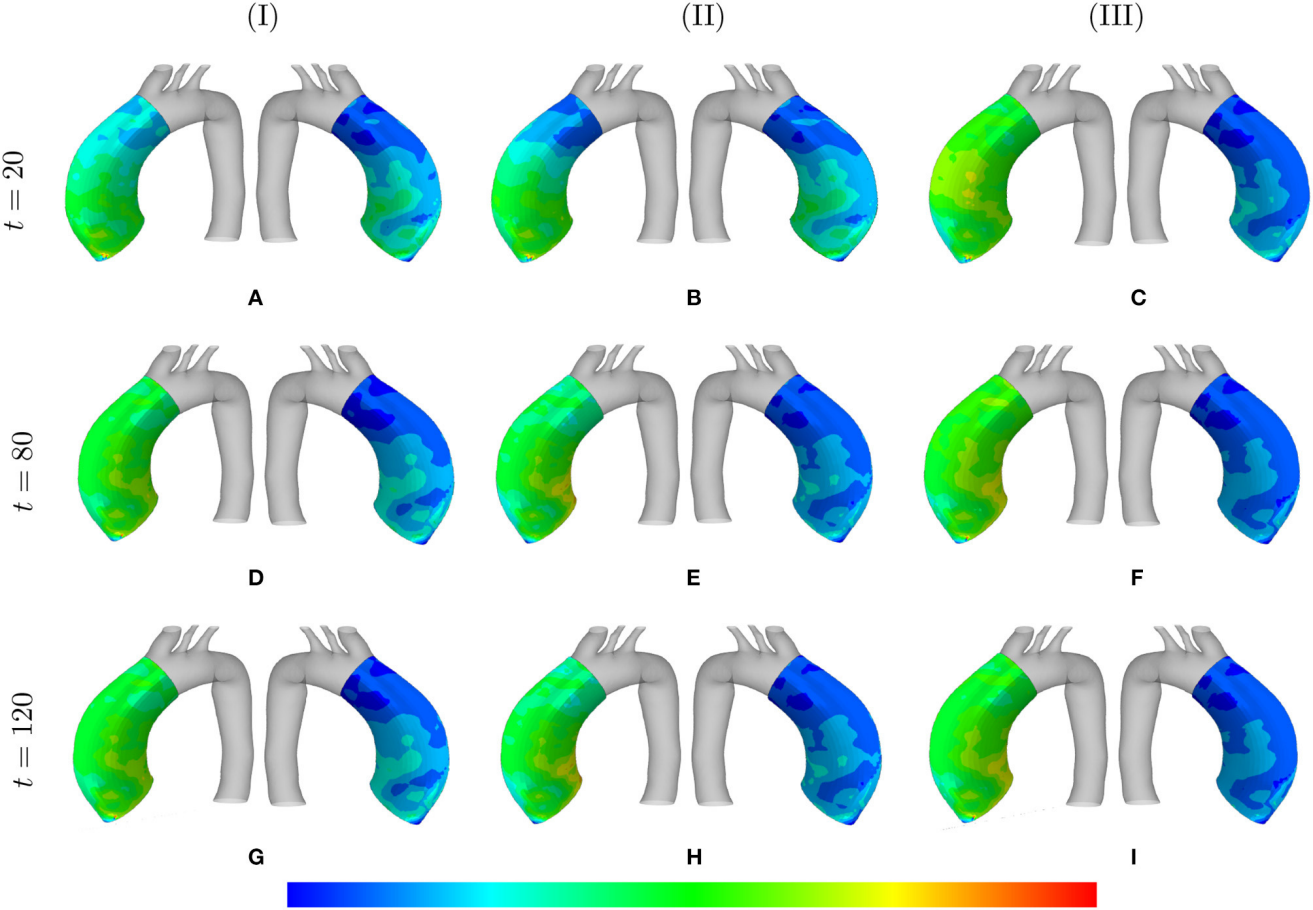
Principal stresses evolution: the results obtained for a two-layer thick-wall patient-specific human ATAA responding to a regional elastin degradation during 15 years. The stress distribution are shown in *t* = {20, 80, 120} [Months] depending on the (column-wise presentation of the) maximum SMCs contractility **(I)** λ_max_ = 1.4; **(II)** λ_max_ = 1.1; and **(III)** λ_max_ = 1.0 for a fixed value of $k_{\sigma }^{{\text{c}}_{j}}=0.05$ and *t*_dam_ = 40 [days]. Each of the columns shows the stress distributions, from left to right, for the adventitia and media layers, respectively. The range of principal stress distributions are **(A)** [6.1e4–5.6e5]; **(B)** [3.7e4–3.8e5]; **(C)** [6.5e4–5.4e5]; **(D)** [6.7e4–7.6e5]; **(E)** [5.8e4–5.1e5]; **(F)** [8.9e4–6.2e5]; **(G)** [6.9e4–8.3e5]; **(H)** [8.3e4–6.6e5]; **(I)** [2.3e5–8.3e5] in [Pa].

The absolute value of the principal stress distributions are shown in Figure 10 for the representative case of $k_{\sigma }^{{\text{c}}_{j}}=0.05$ and *t*_dam_ = 40. The results are compared for different values of the maximum SMCs stretches shown in media and adventitia layers. In fact, the longer G&R occurs, the more stress is transferred from the media to the adventitia layer.

### 3.4. Aneurysm Growth

**Figure 11:**
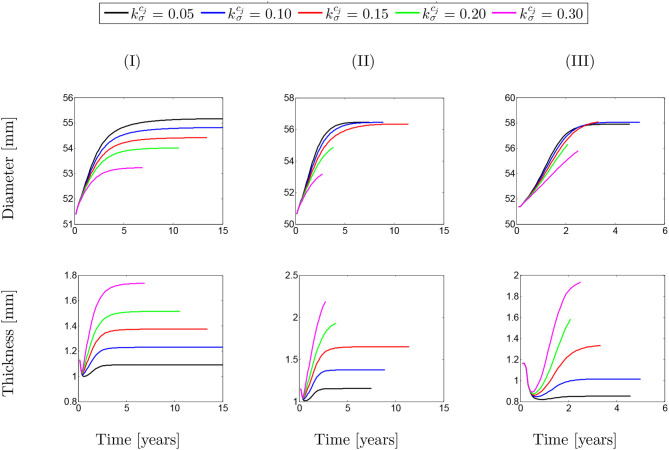
Evolution of arterial diameter with respect to the physical time. The results obtained for a two-layer thick-wall patient-specific human ATAA responding to a regional elastin degradation for 15 years. Different rates of collagen deposition $k_{\sigma }^{{\text{c}}_{j}}/T^{{\text{c}}_{j}}$ are considered depending on the (column-wise presentation of the) maximum SMCs contractility **(I)** λ_max_ = 1.4; **(II)** λ_max_ = 1.1; and **(III)** λ_max_ = 1.0 for a fixed value of *t*_dam_ = 40 [days].

Figure 11 depicts the evolution of the aortic diameter and thickness during G&R. The results are compared for different values of λ_max_ where various rates of collagen deposition are investigated. As expected, faster collagen deposition results in less dilation of the aortic wall and, therefore, faster convergence of the numerical solution. This trend can be observed for both diameter and thickness whose opposite trend is inevitable. In addition, the effects of SMCs maximum contractility, especially in the case of diameter, is evident as a smaller λ_max_ has led to an increase in the diameter. However, this increasing trend of diameter does not completely agree with the thickness of the artery. It seems that thickness decreased in the critical condition (λ_max_ = 1.0).

**Figure 12:**
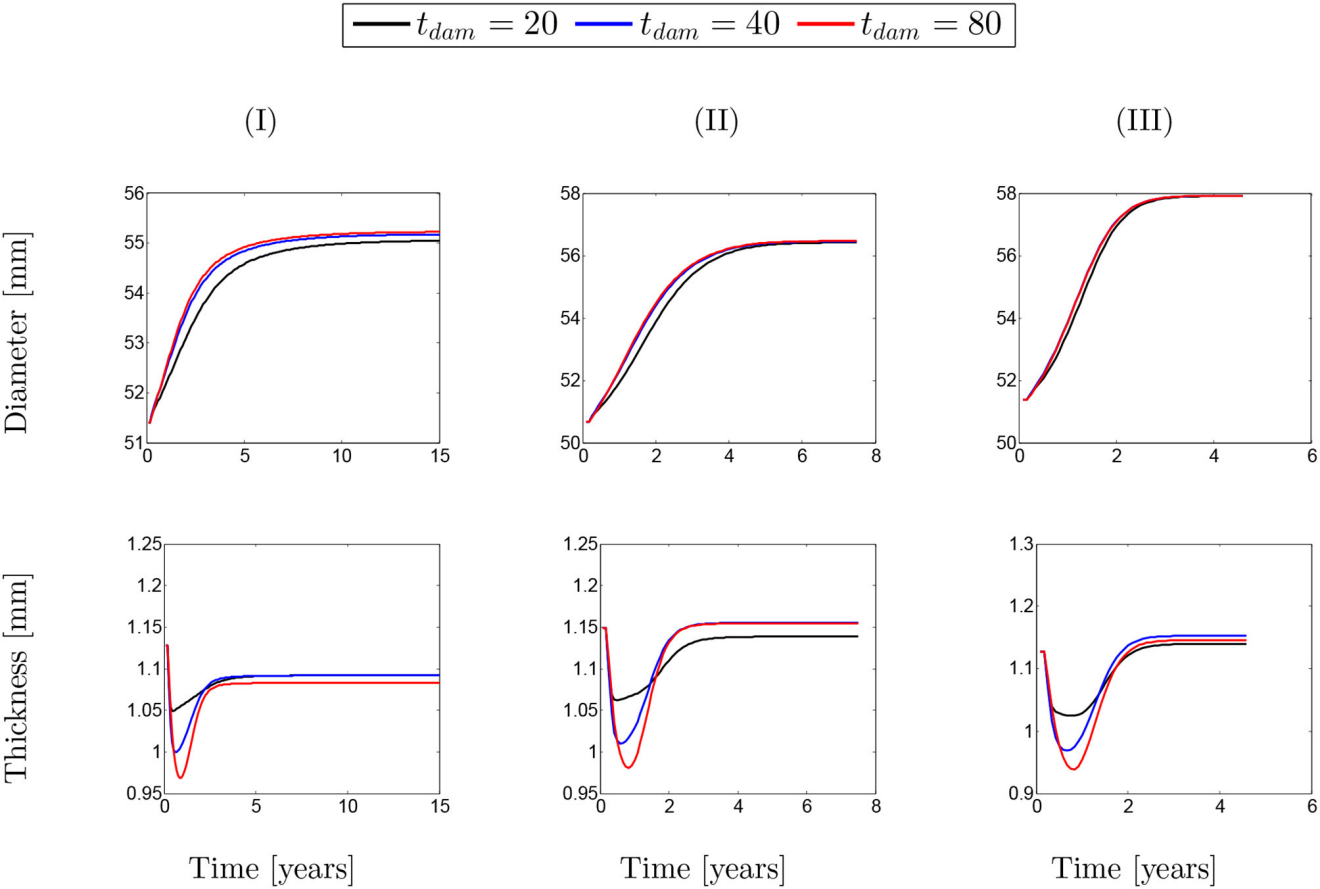
Evolution of artery thickness with respect to physical time *t*. The results obtained for a two-layer thick-wall patient-specific human ATAA responding to a regional elastin degradation for 15 years. Different damage time *t*_dam_ [days] are considered depending on the (column-wise presentation of the) maximum SMCs contractility **(I)** λ_max_ = 1.4; **(II)** λ_max_ = 1.1; and **(III)** λ_max_ = 1.0 for a fixed value of $k_{\sigma }^{{\text{c}}_{j}}=0.05$.

The same behavior can be observed in Figure 12 for different values of damage time. However, it can be generally concluded that whatever the conditions of SMCs active stress, the change in the rate of collagen deposition or damage time does not have a major influence on changing the diameter/thickness of the arterial wall.

**Figure 13:**
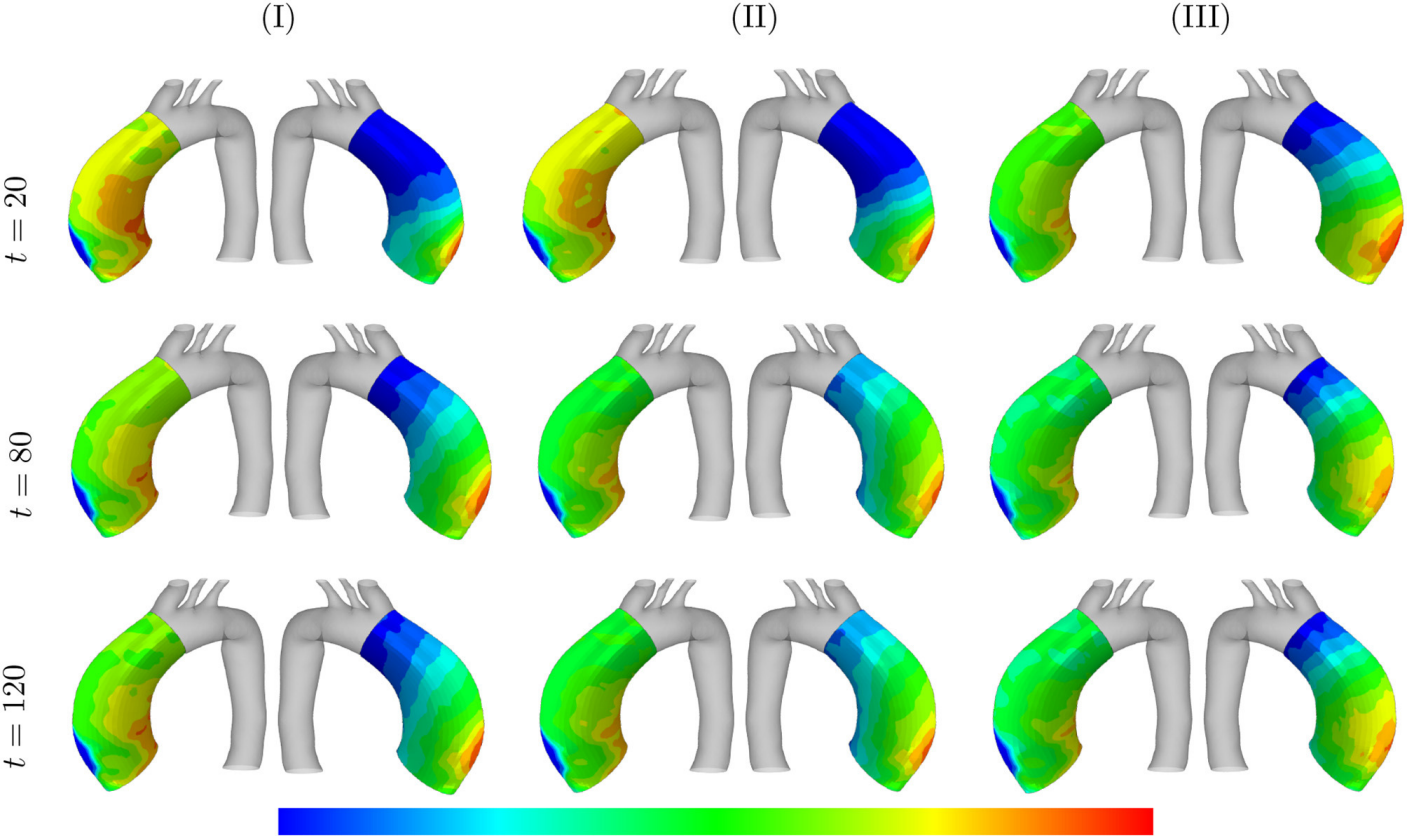
Arterial wall thickness and displacement evolutions: the results obtained for a two-layer thick-wall patient-specific human ATAA responding to a regional elastin degradation during 15 years. The wall thickness distributions are shown at *t* = {20, 80, 120} [Months] depending on the (column-wise presentation of the) maximum SMCs contractility **(I)** λ_max_ = 1.4; **(II)** λ_max_ = 1.1; and **(III)** λ_max_ = 1.0 for a fixed value of $k_{\sigma }^{{\text{c}}_{j}}=0.05$ and *t*_dam_ = 40 [days]. Each column shows, from left to right, the thickness [0.78–1.3] and displacement [5.5e-4–3.5e-3] in [mm], of the arterial wall in the media layers, respectively.

The contour plot of the arterial wall thickness and displacement distributions are shown in Figure 13 for the representative case of $k_{\sigma }^{{\text{c}}_{j}}=0.05$ and *t*_dam_ = 40 in the media layer for different values of maximum SMCs contractility.

**Figure 14:**
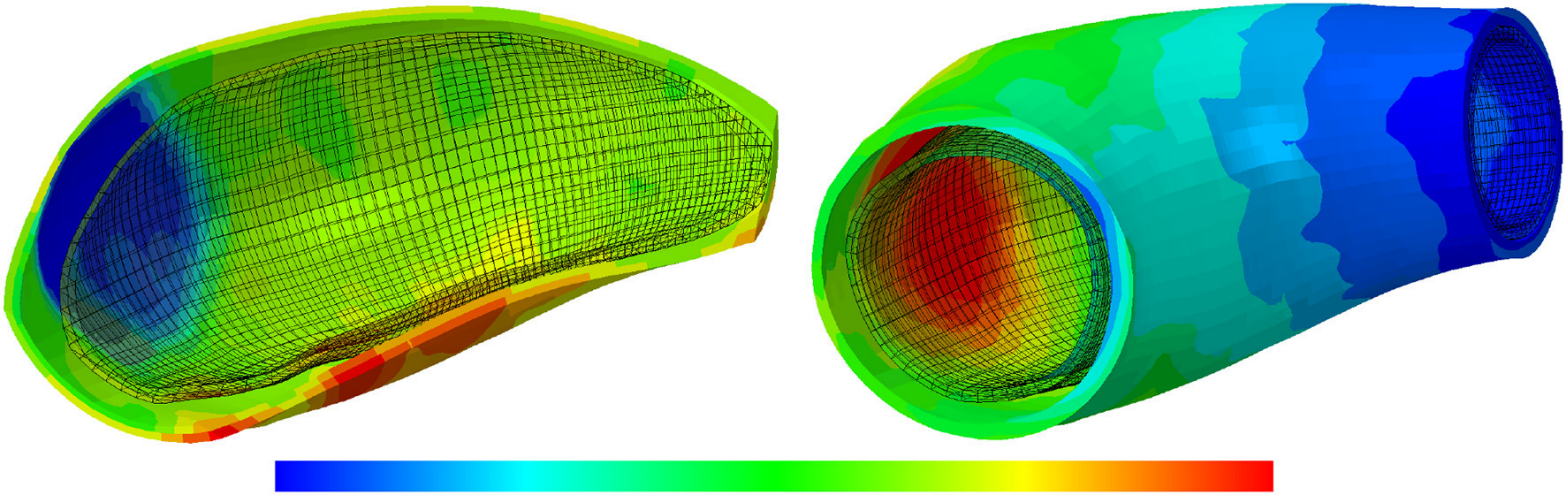
The thickness and displacement distributions of the arterial wall: the results obtained for a two-layer thick-wall patient-specific human ATAA responding to a regional elastin degradation for 15 years. In this representative case, the collagen deposition gain is $k_{\sigma }^{{\text{c}}_{j}}=0.05$, the maximum SMCs contractility is λ_max_ = 1.4 and the damage time is *t*_dam_ = 40 [days]. From left to right, the thickness [0.78–1.3] and displacement [5.9e-4–3.5e-3] in [mm], of the arterial wall are depicted.

Figure 14 also illustrates the arterial wall dilation compared to its initial state through thickness and displacement distributions after 15 years of G&R progression. The maximum displacement and minimum thickness occurred at the location of the localized elastin loss.

## 4. Discussion

In this paper, growth and remodeling of a patient-specific aortic geometry has been computationally modeled using the homogenized CMT in order to predict ATAA evolution while the artery has undergone proteolysis of elastin localized in regions of deranged hemodynamics. To the best of the authors' knowledge, it is the first time that the effects of possible variations in SMC contractility on ATAA progression are simulated in a multi-layer patient-specific model. The results reveal a possible critical condition in which the active stress of SMCs can reduce to zero, which can be interpreted as if SMCs do not pull onto the extracellular matrix anymore, potentially leading to apoptosis.

The paramount importance of the SMCs role in short- and long-term changes of arterial responses is reported in Cox (1975, 1978), Humphrey and Rajagopal (2002), Liu et al. (2008), and Murtada et al. (2012, 2015). The active tension of SMCs, produced over a large range of muscle lengths, can be described through a length-tension relationship (Dorbin, 1973; Rachev and Hayashi, 1999; Gunst et al., 2003; Zulliger et al., 2004; Carlson and Secomb, 2005; Herrera et al., 2005; Syyong et al., 2008; Yamin and Morgan, 2012). In this work, the classic inverted parabola shape is used for the length-tension relationship as it, specifically, describes the overlap between the actin and myosin filaments (Murtada et al., 2012).

Compared to the well-known model proposed by Zulliger et al. (2004), our model, which stems from the work of Murtada et al. (2012), has a number of differences which are:

1. Zulliger et al. (2004) have a multiplicative split between the active and passive stress of SMCs whereas we use an additive split;
2. Zulliger et al. (2004) considered effects of the change of calcium concentration with the stretch, which we disregarded, as these effects mainly manifest for fast deformations, whereas we modeled deformations over large timescales;
3. for the length tension relationship, Zulliger et al. (2004) used a Heaviside function whereas we used an inverted parabola function.

It is worth noting that it is the first time that variations of the active stress are investigated in G&R progression through computational modeling. The situations that we have considered for these variations of the active stress remain partly fictitious because there are no *in vivo* measurements of the active stress *in vivo*.

In this work, a parametric study has been performed on the basis of the time at which the artery is maximally damaged (*t*_dam_), the rate of collagen deposition ($k_{\sigma }^{{\text{c}}_{j}}/T^{{\text{c}}_{j}}$) and the maximum contractility of SMCs (λ_max_) in order to study the effects of active SMCs on G&R of a patient-specific ATAA geometry. Results illustrate the importance of damage time on the expansion rate of the artery such that the larger value of *t*_dam_ leads the higher rate of G&R. Note that elastin degradation during ATAA growth may be related to multiple biological and mechanical parameters including abnormal distribution of Wall Shear Stress (WSS) (Guzzardi et al., 2015) and circumferential stress (Humphrey and Rajagopal, 2002). Our simulations highlight some dependence between the active stress and damage time, especially when the maximum active stretch was larger than the deposition stretch. However, this dependency is dramatically reduced in the other two cases when the maximum SMCs contractility is equal or less than the deposition stretch. The evolution of the SMCs total (active and passive) stresses indicates that the passive contribution is increasing all the time. In fact, increasing both the damage time and maximum SMCs contractility have resulted in an increase in SMCs stresses and in total stresses of the mixture.

It appeared that active stress decreases more when the rate of collagen deposition increases. This makes sense as collagen can take over a part of the stresses borne by SMCs. For instance, in the case of $k_{\sigma }^{{\text{c}}_{j}}=0.05$, the active stress increases and remains at its maximum value since the amount of collagen deposited in the media layer is not sufficient to compensate the loss of active tone in SMCs. This is an opposite trend compared to the case $k_{\sigma }^{{\text{c}}_{j}}=0.30$ in which the fast deposition of collagen prevents the SMCs to reach their maximum contractility and a comparative fast decrease in active stress can be observed. It must be noted that SMC contractility does not dramatically affect the arterial dilation. Considering the evolution of the SMCs total (passive and active) stresses, the monotonically increasing trend of passive stresses can be acknowledged. In some cases (say $k_{\sigma }^{{\text{c}}_{j}}>0.15$), though, the active stresses dominate the behavior of SMCs total stresses. This can also be observed on the evolution of the total mixture stresses which begin to increase once the G&R starts. The decrease in total mixture stresses indicates that if the rate of collagen deposition reaches a certain threshold, the artery becomes thick enough to enable a dramatic reduction of the wall stress. This certainly affects the speed of convergence of the numerical solutions as the computational time was significantly lengthened for these situations.

It must be noted that there are still several limitations and technical challenges associated with the presented model:

- The presented model relies on a number of assumptions. One of these assumptions, stating that initial reference is at homeostasis, is typical of G&R (Kassab, 2008). Although this assumption may be actually satisfied globally at the scale of an organ, we also assume that it is satisfied point-wisely in our G&R model of the arterial wall.
- Considering a uniform thickness for the arterial wall is a limitation of the current work since the distribution of material and structural parameters (e.g., thickness, fiber orientations) of a patient-specific geometry should be consistent with *in vivo* data.
- In this work, the constitutive parameters for the patient-specific model were estimated by curve fitting from the *ex vivo* bulge inflation data of an ATAA segment excised after the surgical intervention of the same patient. However, the *in vivo* material properties of ATAAs (Farzaneh et al., 2019) should be identified non-invasively in clinical applications.
- We have generated fictitious situations where the active stretch of SMCs evolves with the actual stretch of the tissue and have echoed this evolution onto the active stress through the inverted parabola function. Although these situations can be justified if SMC proliferate at an extremely low rate and do not adapt to aneurysm progression, they remain fictitious and there is a pressing need to characterize the evolution of SMCs length-tension relationships in aortic aneurysms *in vivo* in order to improve the predictions of G&R computational models.
- Several biological pathways may be activated during aneurysm development and induce adaptation mechanisms of SMCs that were not considered in this work. More specifically, the distribution of WSS is complex in ATAAs (Condemi et al., 2017) and this may have an impact on SMCs which should be included in future developments of the model.

## 5. Conclusion

In this work, the contribution of SMCs contractility during aneurysmal growth and remodeling has been computationally investigated with a multi-layer patient-specific model of the human aorta subjected to localized elastin degradation. The model relies on the homogenized constrained mixture theory. The results show that the active contribution of SMCs is of paramount importance in analyzing the total stress field of the mixture. It, in fact, affects the rates of collagen deposition by which the elastin loss is gradually compensated. Interestingly, a critical condition has been observed, under which the active stress of SMCs could drop and possibly result in apoptotic conditions. Future work will carry on investigating the major role of SMCs contractility on G&R in aneurysm development, considering supplemental biological pathways in our computational model in order to account for actual adaptation mechanisms undergone by SMCs.

## Data Availability Statement

The original contributions presented in the study are included in the article/supplementary material, further inquiries can be directed to the corresponding author/s.

## Ethics Statement

The studies involving human participants were reviewed and approved by CHU Saint-Etienne. The patients/participants provided their written informed consent to participate in this study.

## Author Contributions

AG: software implementation, simulations, validation, analysis of results, and writing—original draft presentation. All authors supervision of the research, methodology, funding, analysis of results, reviewing, and editing of manuscript.

## Conflict of Interest

The authors declare that the research was conducted in the absence of any commercial or financial relationships that could be construed as a potential conflict of interest.
